# Malaria-derived hemozoin skews dendritic cell responses to bacterial infections by reducing interferon gene-transcription by SWI/SNF-NuRD

**DOI:** 10.1016/j.isci.2025.113046

**Published:** 2025-07-03

**Authors:** Gintare Lasaviciute, Kanwal Tariq, Anaswara Sugathan, Jaclyn Quin, Mareike Polenkowski, Ioana Bujila, Oleksii Skorokhod, Marita Troye-Blomberg, Eva Sverremark-Ekström, Ann-Kristin Östlund Farrants

**Affiliations:** 1 Department of Molecular Biosciences, The Wenner-Gren Institute, Stockholm University, Stockholm, Sweden; 2 Department of Life Sciences and Systems Biology, University of Torino, Torino, Italy

**Keywords:** Immune response, Molecular mechanism of gene regulation, arasitology

## Abstract

Hemozoin (HZ), the malaria pigment, is associated with the disease when released during the pro-inflammatory blood stage and co-infections with bacteria lead to a more severe disease progression. The underlying mechanisms are poorly understood and, here, we show that the impact of co-exposure to HZ and lipopolysaccharide (LPS) on monocyte-derived dendritic cells (moDC) alters the early transcriptional response to a subset of IFNγ controlled genes, HLA-DR, and PD-L1. HZ-exposure had no effect on inflammatory genes, which were substantially induced by LPS. The reduced expression of HLA-DR and PD-L1 by HZ was associated with the chromatin remodeling complex NuRD and a decreased binding of the NF-κB transcription factor RELA compared to cells stimulated with LPS alone. NuRD replaced the SWI/SNF complex variant PBAF at the specific promoters, without chromatin accessibility changes. The immune modulatory effect of HZ may lead to changed immune responses to bacterial co-infections.

## Highlights

• Hemozoin dampens the early acute response of LPS by impeding a subset of ISG promoters
• LPS activates ISG through the SWI/SNF complex PBAF, HZ represses the activation through NuRD
• Inflammatory genes are activated through the SWI/SNF complex ncBAF and not affected by HZ
• HZ activates NF-κB and IRF3, keeping DC in an immature state by maintaining DC SIGN and CD206

## Graphical abstract

**Figure:**
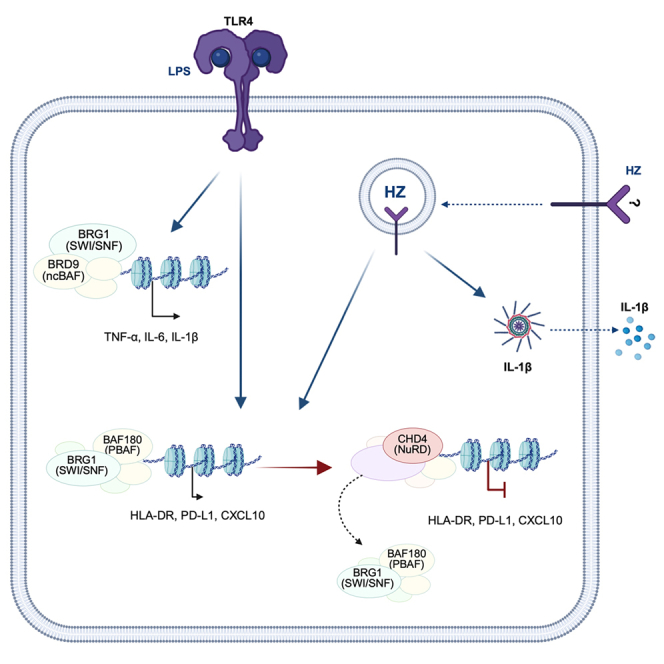

## Teaser

Immune response; Molecular mechanism of gene regulation; Parasitology

## Introduction

The human immune response to malaria is multifaceted and complex since the malaria parasite, Plasmodium, has many life stages and infect several organs. During the blood stage, the infected erythrocyte releases the parasite merozoites together with erythrocyte and malaria specific contents, which are quickly endocytosed by innate immune cells and subsequently accumulate in tissues. The parasite release elicits a strong innate pro-inflammatory response, which leads to the activation of T-cells and to the clearance of the parasite.^1^ Co-infections with viruses or bacteria usually accelerate both the malaria and the infection, in particular in children and naive infected individuals.^2^^,^^3^ How the released Plasmodium parasite and host molecules^3^^,^^4^^,^^5^ contribute to the more sever disease progression in co-infections is poorly understood.

The pro-inflammatory response to *P. falciparum* is correlated not only with the release of the parasite^6^ but also with parasite-derived products, such as hemozoin (HZ), a crystalline pigment from Plasmodium digested heme, and its phagocytosis by innate cells.^7^^,^^8^^,^^9^^,^^10^ The acute pro-inflammatory response, characterized by the release of cytokines and chemokines, such as TNF-α, IL-6, and CXCL10, is followed by a dampening of the pro-inflammatory phase, involving IL-10^8^^,^^11^ and TGFβ.^12^ Studies of *in vitro* stimulated PBMCs and isolated innate cells, such as monocyte/macrophages or dendritic cells (DCs), incubated with either infected red blood cell lysate (iRBC) or purified HZ, show that they do not elicit a pro-inflammatory response^13^^,^^14^ unless at high concentration.^15^ Other stimuli are necessary for the response and isolated macrophages, monocytes and DCs require external ROS to mount a pro-inflammatory response.^16^^,^^17^ Nevertheless, iRBC and HZ exposures affect immune innate cells in other ways; monocytes/macrophages and DCs display reductions of the immune receptor MHCII and costimulatory factors CD83 and CD86, important for T cell activation.^7^^,^^8^^,^^18^^,^^19^ In particular, DCs which are antigen-presenting cells with the ability to also activate naive CD4 T-cells, are the major instigator of a dysregulated T cell activation during infections. iRBC or HZ stimulation activates a few immune gene, but induces a transcriptional change in other pathways; short-term exposure of blood DCs to iRBC activates lipid synthesis-related genes, probably through the PPARγ pathway^14^ and induces the expression and the secretion of malaria associated chemokines.^13^^,^^14^ HZ exposure activates a subset of DC-specific genes correlated to a partial maturation phenotype^19^ as well as PPARγ targets genes.^7^ In addition, HZ exposure impairs DC function at several stages; compromising the differentiation of monocytes to DC (moDC) *in vitro*^7^^,^^20^ and impairing moDC maturation upon exposure to stimuli^7^^,^^9^^,^^15^^,^^19^^,^^20^^,^^21^ causing impaired T cell activation.^9^^,^^15^^,^^20^^,^^21^^,^^22^

Malaria is suggested to be immunosuppressive and both iRBC and HZ have been shown to lead to a reduced response to a second infection,^13^^,^^14^^,^^15^^,^^16^^,^^17^^,^^18^^,^^19^^,^^20^^,^^21^^,^^22^^,^^23^^,^^24^^,^^25^ possibly by inducing an innate tolerance memory response. DCs pre-exposure to iRBC or HZ display an impaired maturation to subsequent stimulations with LPS.^7^^,^^15^ However, monocytes from naive blood donors elicit a higher response upon a second stimulus when pre-exposed to iRBC and HZ, suggesting a trained phenotype.^26^^,^^27^ A trained or tolerized phenotype is established by epigenetic reprogramming, such as an increased level of the histone modifications H3K4me3 and H3K27Ac at specific genes loci, in addition to metabolic reprogramming.^28^^,^^29^ These changes rely on long-term exposure, at least for 24 h for the first stimulus, followed by a rest-period before subsequent stimulations with related or unrelated pathogens.^28^^,^^29^ Short-term exposure of malaria antigens is also likely to have modulatory effects and alter the response to pathogens, important in endemic areas with many infectious diseases coexisting.^30^ The poor initial inflammatory response of innate cells to HZ or iRBC is still associated with the early activation of NF-κB,^7^^,^^31^ however, in an MYD88-independent way.^24^^,^^32^^,^^33^ This raises the question of whether iRBC and HZ have immunomodulatory effects that alter the early acute response to subsequent or simultaneous stimulation with pathogens.

To elucidate the mechanism behind the early immune suppressing effect, we exposed human moDCs, differentiated from malaria-naive blood donors, to HZ opsonized with human serum together with LPS as a strong bacterial pathogen-associated molecular patterns (PAMP). We show that exposure to HZ modulated the acute transcriptional response to LPS; specifically, co-exposure with HZ prevented LPS-induced HLA-DR and PD-L1 gene expression, as well as CXCL10, in contrast to inflammatory genes, where HZ had no effect on the LPS-induced activation. Many immune genes are regulated by NF-κB and IRFs in an SWI/SNF dependent manner with specific constellations operating on different gene promoters and enhancers.^34^^,^^35^^,^^36^ We show that the HLA-DR and PD-L1 promoters were associated with the BAF180-PBAF variant upon LPS stimulation, and this complex was replaced by the silencing NuRD complex in co-exposed cells. NuRD has been shown to antagonize the SWI/SNF on the promoter and enhancer of some inflammatory genes during stimulation, but first during the late early phase.^37^ Importantly, despite the association of chromatin remodeling factors, the regulation of these genes was not dependent on chromatin alterations. The genes involved in the moDC response either established an open configuration during differentiation from monocytes to moDCs, or were already pre-set in an open state at an even earlier stage, as observed for enhancers of mouse T-cells.^38^ HZ also activated IRF3, which was found to bind together with RELA to the promoters of DC SIGN (CD209) and CD206 (MCR1), important to actively maintain their transcription to keep moDCs in an immature state.^39^ We propose that HZ alters specific gene promoters, resulting in a changed response to simultaneous infections with bacteria. Since species differences in effectors for signaling pathways and cell type responses exist between mouse and man,^40^^,^^41^^,^^42^ we suggest that in human cells, the modulation occurs through the association of either SWI/SNF complexes or the NuRD complex early during an infection, which leads to a capacity to transient alterations of subsequent T cell response. These results demonstrate that HZ modulates the human early acute response, and are potentially important for clinical treatment strategies and vaccination programs.

## Results

### HZ compromises LPS-induced upregulation of HLA-DR and PD-L1 gene expression

**Figure 1:**
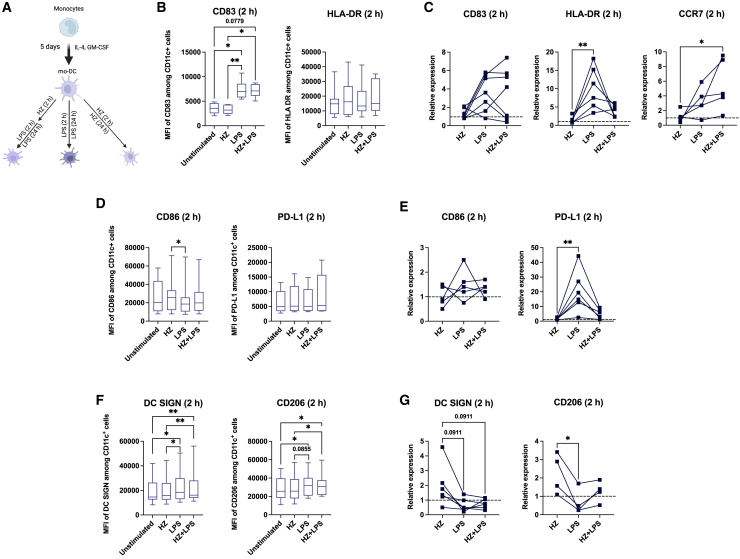
HZ hampers the upregulation of HLA-DR and PD-L1 transcripts during co-exposure with LPS (A) Experimental model of monocytes differentiation to moDCs during 5 days culture and exposure to different stimuli. MoDCs were pre-exposed to HZ for 2 h, then LPS was added for additional 2 h or 24 h (co-exposure condition). Cells kept in complete culture medium served as the control. (B) The mean fluorescent intensity (MFI) of CD83 and HLA-DR in moDCs after 2 h of exposure, *n* = 7. (C) The relative mRNA expression of CD83, HLA-DR, and CCR7 in moDCs after short-term exposure. Each line represents samples from the same donor. The expression is related to unstimulated cells, represented by the dotted line, *n* = 5–7. (D) The mean fluorescent intensity (MFI) of CD86 and PD-L1 in moDCs after 2 h exposure, *n* = 5–7. (E) The relative mRNA expression of CD86 and PD-L1 after short-term exposure. Each line represents samples from the same donor, *n* = 5–6. (F) The mean fluorescent intensity (MFI) of DC SIGN and CD206 after 2 h of exposure, *n* = 12. (G) The relative mRNA expression of DC SIGN and CD206 after short-term exposure. Each line represents samples from the same donor, *n* = 4–6. (B, D, F) Boxplots cover data between the 25th and the 75th percentile with median as the central line and whiskers showing min-to-max. (B–G) Paired Friedman test followed by Dunn’s multiple comparison was used to determine statistical difference, *n*.s. = *p* > 0.05, ∗*p* < 0.05, ∗∗*p* < 0.01.

HZ released during the blood stage is one of the pathogens that contribute to long-term malaria disease symptoms and may induce a trained immune phenotype,^26^^,^^27^ but the effects of short-term exposure are less understood. To address the question whether HZ has an impact on the early acute moDC phenotype or the functional response to an unrelated microbial compound, we pre-incubated moDCs with HZ for 2 h to allow its phagocytosis before adding LPS, as depicted in Figure 1A. To mimic the state of HZ after its release in the bloodstream, we used an opsonized preparation in which the HZ crystal contained plasma components dissolved in PBS, and LPS stimulation of unstimulated cells was a control for LPS induction. HZ was phagocytosed by the cells already after 2 h, as shown by the increased granulation of cells observed by flow cytometry (Figure S1A), in accordance with previous reports.^7^^,^^9^^,^^10^^,^^19^ We next investigated the role of HZ on moDC maturation after 2 h exposure. Exposure of HZ alone did not induce moDC maturation, as the expression of HLA-DR and the maturation marker CD83 was not elevated, nor did HZ interfere with the LPS-induced maturation, as the expected elevation of CD83 was not reduced in co-exposed cells (Figure 1B). The process of maturation depends on the activation of different markers in a time-dependent manner with some factors relying on an early transcriptional response. This led us to investigate mRNA levels of these DC markers upon HZ and LPS-stimulation. Because of the large variation in response in samples from healthy blood donors due to the genetic makeup and health history, we present the transcriptional response in the treated samples to each individual. HZ alone did leave transcription of the factors unaltered, but it did reduce the LPS-triggered increase in HLA-DR transcripts when the cells was co-exposed to LPS and HZ (Figure 1C). Transcription of CD83, however, was not significantly changed by any treatment, whereas CCR7, a receptor transcriptionally induced during DC maturation, was significantly induced by co-exposure, showing that HZ affected transcription in a gene-specific manner (Figure 1C). Both HLA-DR and CD83 proteins were clearly upregulated after 24 h in cells stimulated with LPS alone or co-exposed to LPS and HZ in comparison with unstimulated cells (Figure S1B), indicating that the impact was transient.

HLA-DR is involved in T cell activation and the early transcriptional reduction of HLA-DR prompted us the investigated other co-stimulatory receptors, CD86 and PD-L1, involved in T cell regulation. Similar to HLA-DR, no change of the surface expression of CD86 and PD-L1 occurred in any treatment at 2 h, apart from a down regulation of CD86 in LPS stimulated cells compared to cells exposed to HZ (Figure 1D). Nevertheless, the mRNA levels of PD-L1 displayed a similar pattern to that of HLA-DR; co-exposure with HZ strongly reduced the expression of LPS-induced PD-L1 (Figure 1E). The level of CD86 mRNA did not differ among samples at this early time point (Figure 1E). Similar to HLA-DR, the level of PD-L1 at the surface were increased after 24 h compared to unstimulated cells (Figure S1C), suggesting that differences in the early transcriptional response have little consequence at later time points. We therefore investigated whether the transcription of HLA-DR and PD-L1 genes increased at later time points and we detected a delayed response; HLA-DR mRNA was induced at 12 h and PD-L1 mRNA was induced at 6 h compared to 2 h for LPS alone, and also then at lower levels. (Figure S1D, top and middle panel). In human cells, HLA-DR and PD-L1 are under IRF1 control,^41^^,^^42^ which led us to investigate whether this factor increased later and therefor activated the transcription during co-stimulation at later time points. The transcriptional response of IRF1 was delayed in co-exposed cells, similar to HLA-DR and PD-L1; it was induced at 6 h instead for 2 h as in LPS exposed cells alone (Figure S1D, bottom panel). This indicates that the induction by LPS is compromised by HZ mainly at the early time points, and that the later response is sufficient for the surface expressed proteins to reach the same level in co-exposed cells as in LPS stimulated cells alone after 24 h.

To assess if moDCs remained immature upon HZ addition, we investigated the surface expression of the C-type lectin receptors DC SIGN and CD206, which are associated with an immature phenotype.^39^ Both receptors displayed a slight increase in LPS and co-exposed cells after 2 h of exposure (Figure 1F), whereas a clear downregulation in these samples was observed after 24 h (Figure S1E). This downregulation was reflected at the transcriptional level early acute phase in cells exposed to LPS and co-exposed to LPS and HZ, with both treatments showing a trend toward a reduced expression compared to the cells only exposed to HZ (Figure 1G). Taken together, our results suggest that HZ interferes with the early transcriptional LPS activation of a subset of DC maturation genes, and maintains the transcriptional level of DC immaturity genes.

### HZ does not influence the secretion or gene expression of LPS-induced inflammatory cytokines

**Figure 2:**
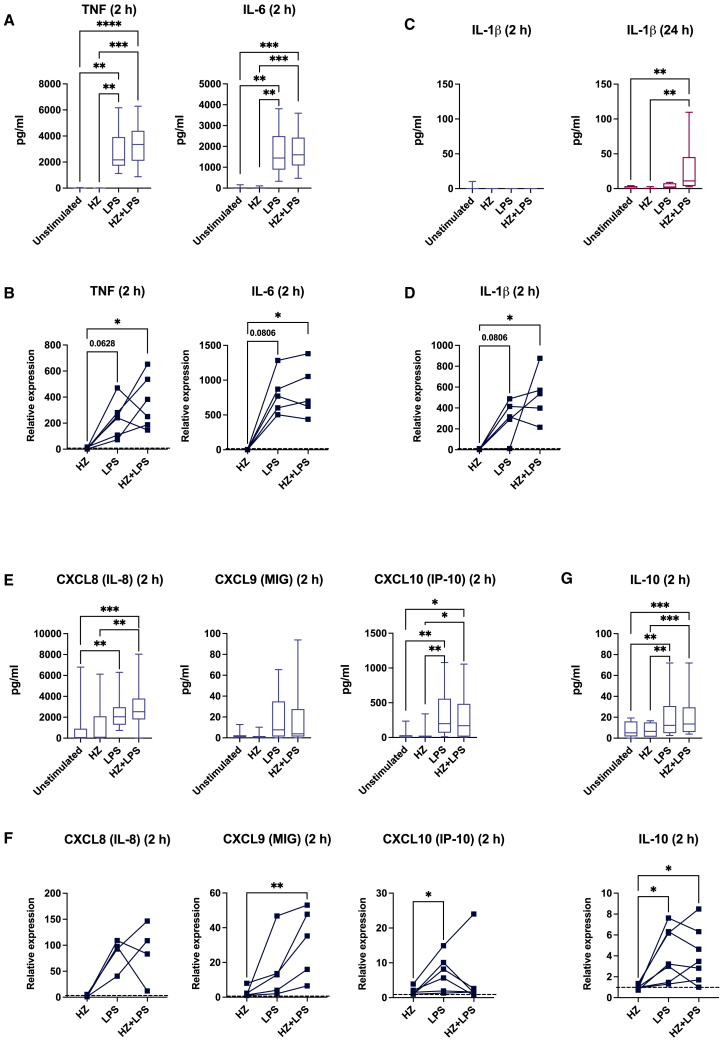
HZ does not interfere with the upregulation of inflammatory gene transcripts, but both HZ and LPS are required for the release of inflammasome-derived IL-1β (A) Soluble levels of TNF-α and IL-6 in moDCs after 2 h exposure, *n* = 12. (B) Relative mRNA expression of TNF-α and IL-6. Each line represents samples from the same donor. The expression in the treated samples is related untreated cells, presented as a dotted line, *n* = 5–6. (C) Soluble levels of IL-1β after 2 h and 24 h exposure, *n* = 9–12. (D) Relative mRNA expression of IL-1β after 2 h of exposure. Each line represents samples from the same donor. The expression in the treated samples is related to the level in unstimulated, represented as a dotted line, *n* = 5. (E) Soluble levels of CXCL8, CXCL9, CXCL10 after 2 h exposure, *n* = 14. (F) Relative mRNA expression of CXCL8, CXCL9, CXCL10 after 2 h of exposure. Each line represents samples from the same donor. The expression is related to unstimulated cells, presented as a dotted line, *n* = 4–6. (G) Soluble levels, upper panel, and relative mRNA expression, lower panel, of IL-10 after 2 h exposure. Each line represents samples from the same donor. The expression is related to unstimulated cells, presented as a dotted line, lower panel, *n* = 7–12. (A, E, G) Boxplots cover data between the 25th and the 75th percentile with median as the central line and whiskers showing min-to-max. (A–G) Paired Friedman test followed by Dunn’s multiple comparison was used to determine statistical difference, n.s. = *p* > 0.05, ∗*p* < 0.05, ∗∗*p* < 0.01, ∗∗∗*p* < 0.001, ∗∗∗∗*p* < 0.0001.

We next investigated whether HZ had an impact on the acute inflammatory and anti-inflammatory response induced by LPS. LPS induced the secretion of the primary response cytokines TNF-α and IL-6 as early as after 2 h, and IL-23 secretion after 24 h, regardless whether HZ was present or not (Figures 2A and S2A). IL-12 was not secreted in any samples after 2 h (not shown). The inflammatory transcriptional response to LPS was also seen in the TNF-α, IL-6, and IL-23 genes after 2 h and this response was not interfered with by HZ (Figures 2B and S2A, lower panel). LPS alone and co-exposure to LPS and HZ also induced a clear transcriptional response of the early primary gene IL-1β, but the protein was only secreted in co-exposed cells after 24 h (Figures 2C and 2D). This is in agreement with a two-step mechanism relying on transcriptional activation of key genes in the pathway and on the assembly of the inflammasome.^43^^,^^44^ LPS induced the transcription of the inflammasome component NLRP3 and a similar trend was observed in caspase 1 (Figure S2B). The inflammasome activation involves the cleavage of pro-caspase 1 and we did not detect any cleavage at the 2 h time point after stimulation, showing that the transcriptional induction was insufficient for the full activation of the inflammasome to release IL-1β (Figure S2C). During inflammation, the production of IL-1β is balanced by the IL-1 receptor antagonist (IL-1RA)^45^ and the secretion of the IL-1β antagonist was significantly secreted in co-exposed cells at 24 h, after an initial reduction at 2 h. The mRNA expression at 2 h was not changed in any treatment (Figure S2D).

### HZ does not influence the secretion or gene expression of LPS-induced chemokines or anti-inflammatory cytokines

Chemokine release is also associated with the malaria blood stage, but HZ alone did not induce the secretion of any of the chemokines investigated, CXCL8 (IL-8), CXCL9, CXCL10 (Figure 2E), CCL2 and CCL5 (Figure S2E, upper panel). Similar to other pro-inflammatory genes, HZ did not interfere with the LPS-induced secretion of chemokines (Figure 2E), nor with the transcriptional activation of chemokines, except for CXCL10 (Figures 2F and S2E, lower panel). The transcriptional response of CXCL10 resembled the pattern of HLA-DR and PD-L1; the increase was only detected in LPS exposed cells but not in LPS and HZ co-exposed cells (Figure 2F). We also assessed anti-inflammatory cytokines and HZ did not interfere with the anti-inflammatory response, as LPS-induced IL-10 production was maintained at both the protein and transcriptional levels 2 h post exposure (Figure 2G). Furthermore, HZ did not affect the gene expression of the immunosuppressive or tolerance markers SOCS2 and IDO1, or change the LPS response in co-exposed cells (Figure S2F). We also investigated the protective enzyme heme oxygenase (HO-1), as it is induced upon haem release and dampens the immune response.^46^ HZ exposure did not trigger the gene expression of HO-1, regardless of the presence of LPS (Figure S2F, lower panel). In summary, HZ did not induce an inflammatory response, or interfere with the response to LPS regarding secretion of cytokines and chemokines, nor with the transcriptional induction of these genes.

### TLR4 activation by LPS is not affected in the presence of HZ

**Figure 3:**
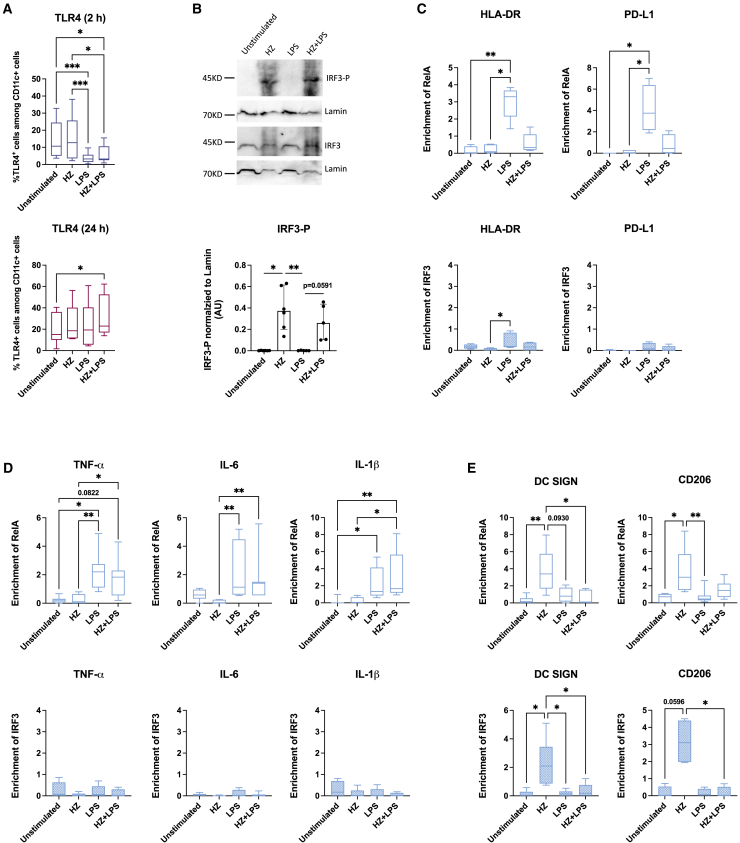
HZ influences transcription factor recruitment to the promoters of moDC genes after short-term exposure (A) The percentage of TLR4 expressing cells among CD11c^+^ moDC population. Paired Friedman test followed by Dunn’s multiple comparison was used to determine statistical difference, ∗*p* < 0.05, ∗∗∗*p* < 0.001. *n* = 6–12. (B) Representative immunoblots of the phosphorylated IRF3 and the IRF3 protein, with immunoblots of lamin shown. The MW are depicted at the left of the blots. IRF3 levels were normalized to the loading control β-lamin levels. The quantitative analysis of the IRF3-P is shown in the bottom panel as scatter dot plots, with individual data points shown as circles and bars representing the median with interquartile range, *n* = 5–6. (C) The enrichment of RELA, top panel, and IRF3, bottom panel, at the promoters of HLA-DR and PD-L1, *n* = 4–6. (D) The enrichment of RELA, top panel, and IRF3, bottom panel, at the promoters of TNF-α, IL-6 and IL-1β, *n* = 4–9. (E) The enrichment of RELA, top panel, and IRF3, bottom panel, at the promoters of DC SIGN and CD206, *n* = 4–8. (A, C, D, E) Boxplots cover data between the 25th and the 75th percentile with median as the central line and whiskers showing min-to-max. (B–E) Non-parametric Kruskal-Wallis ANOVA test, followed by Dunn’s multiple was comparison was applied to determine significant differences, n.s. = *p* > 0.05, ∗*p* < 0.05, ∗∗*p* < 0.01. The data are present as median with interquartile range.

To investigate the mechanism behind the dampening effect of HZ on the transcriptional response of HLA-DR and PD-L1 but not on inflammatory genes, we examined the induction of signaling pathways and activation of transcription factors. LPS-bound TLR4, which signals early in the acute phase through the NFkB pathway, undergoes internalisation into the endosome and the response depends on the strength of the signal triggered, which leads to differential downstream effects.^47^^,^^48^ The percentage of cells expressing TLR4 at the surface was not affected by the exposure to HZ; a significant decrease occurred after 2 h both in cells stimulated with LPS and cells exposed to LPS and HZ (Figure 3A) and the receptor level was restored at 24 h, even significantly upregulated in co-exposed cells (Figure 3A). We conclude that variation in the activation of TLR4 was unlikely to cause modulations of the gene expression by HZ in co-exposed cells. A receptor for HZ is still not identified, but CLEC12A, the receptor for uric acid has been suggested to be the receptor for phagocytosis of HZ.^49^ The surface level of CLEC12A did not change in any treatments at 2 h or 24 h (Figure S3A).

The TLR4 ligation by LPS activates the NF-κB transcription factor RELA/p65 in a MYD88 dependent way, whereas it is not clear whether HZ activates TLR4^24^^,^^32^^,^^33^ but it activates NF-κB in an MYDD88 independent manner.^24^^,^^32^^,^^33^ In addition to NF-κB, IRF3 activation is an early response to viruses in human cells and is involved in the induction of several immune genes in human DCs in response to a viral infection,^50^^,^^51^ which led us to investigated its activation in response to HZ and LPS. IRF3 was activated, as shown with the presence of phosphorylated IRF3, in cells exposed to HZ alone and cells co-exposed with LPS and HZ (Figure 3B), showing that HZ but not LPS stimulation resulted in an early IRF3 activation. The ratio between phosphorylated IRF3 and the IRF3 protein in each sample gave a significant difference with HZ alone, with lower activation in samples co-stimulated by HZ and LPS, indicating that the LPS compromises the activation (Figure S3B, lower panel).

### HZ phagocytosis changes the transcription factor recruitment to promoters

To investigate the mechanism behind the differential gene response in co-exposed cells at 2 h, we examined the binding of RELA and IRF3 to the promoters of HLA-DR and PD-L1 genes and compared their binding to pro-inflammatory gene promoters. The chromatin used was sheared to approximately 150–250 bp DNA-fragments in all treatments (Figure S3C). In line with the higher gene expression of HLA-DR and PD-L1 in LPS-stimulated cells, their promoters showed significant RELA binding in cells stimulated with LPS alone, while HZ and LPS co-exposed cells displayed markedly lower levels of binding (Figure 3C, upper panel). Despite being activated by HZ, IRF3 was only recruited at low levels to these promoters (Figure 3C, lower panel), indicating that these genes are not targeted by IRF3 at this early time point. In contrast to the effect on HLA-DR and PD-L1, HZ did not hamper the binding of RELA at the promoters of inflammatory genes in LPS and HZ co-exposed cells, as it was significantly recruited to the promoters of the primary response gene TNF-α and the secondary response genes IL-6 and IL-1β, to the same extent as in cells stimulated by LPS alone (Figure 3D, upper panels). A similar trend in RELA binding was also observed for IL-23 and CCL5 genes (Figure S3D). IRF3 did only bind at low non-significant levels to the promoters of the inflammatory genes (Figure 3D, lower panels), even to the IRF3 target gene CCL5 after 2 h (Figure S3D). However, both RELA and IRF3 were bound to the IL1-RA gene promoter, which is an IRF3 target gene in human DCs,^41^ in HZ exposed cells, but not in co-exposed cells (Figure S3E), showing that the activated IRF3 was able to bind to some of its target genes.

The slightly lower mRNA levels of DC SIGN and CD206 in LPS stimulated cells compared to HZ exposed cells (Figure 1F), prompted us to also assess transcription factor binding at their promoters. Furthermore, the published RNA-seq dataset from mDCs to iRBC shows an induction of DC SIGN by iRBC, whereas the transcript level in LPS-exposed cells remained at the unstimulated level.^14^ We detected both RELA and IRF3 binding to the promoters of DC SIGN and CD206 in cell exposed to HZ alone (Figure 3E), a binding that was counteracted in co-exposed cells (Figure 3E). The binding of factors did not induce a transcriptional response of the genes, but prevented the slight downregulation, which indicates that it instead actively maintained the mRNA levels of DC SIGN and CD206 to keep the cells in an immature state. Taken together, HZ not only impairs the recruitment of activating factor at certain gene promoters in response to LPS, but also affects other groups of gene to maintain the level of transcription, while leaving the regulation of these genes by other co-stimulations unchanged.

### Chromatin states are established during differentiation and not during maturation

**Figure 4:**
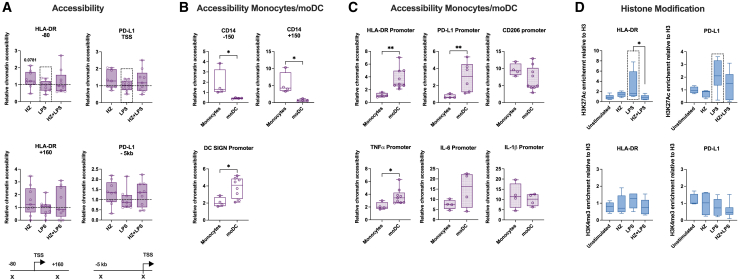
Exposure to HZ or LPS does not alter chromatin state at the promoters of moDC genes (A) Relative chromatin accessibility at the promoters of HLA-DR and PD-L1 genes, top panel, and an identified enhancer for PD-L1. The accessibility is related to that of IL-2 gene promoter. The dotted box represents the samples with altered gene expression. The primer positions are depicted under the graphs for each gene. Non-parametric Wilcoxon matched-pairs signed rank test was applied to determine significant differences, n.s. = *p* > 0.05, *n* = 9. (B) Relative chromatin accessibility at the promoters of CD14, measured before and after the TSS, top panel, and DC SIGN, bottom panel, in isolated monocytes and differentiated moDCs on day 5. Non-parametric Mann-Whitney test was used to determine the differences, ∗*p* < 0.05, *n* = 4. (C) Relative chromatin accessibility at the promoters of HLA-DR, PD-L1, CD206, TNF-α, IL-6 and IL-1β, in isolated monocytes and differentiated moDCs on day 5. Non-parametric Mann-Whitney test was used to determine the differences, ∗*p* < 0.05, ∗∗*p* < 0.01, *n* = 4–9. (D) The recruitment of histone H3K27Ac (upper panels) and histone H3K4me3 (lower panels), at the promoters of HLA-DR and PD-L1 genes. H3K27Ac and H3K4me3 were normalized to the enrichment of histone H3. (A–D) Boxplots cover data between the 25th and the 75th percentile with median as the central line and whiskers showing min-to-max. Non-parametric Kruskal-Wallis ANOVA test, followed by Dunn’s multiple was comparison was applied to determine significant differences, n.s. = *p* > 0.05, ∗*p* < 0.05, *n* = 4–8.

The differential binding of RELA and IRF3 to gene promoters in exposed cells motivated us to investigate the chromatin states at these promoters. However, no significant changes were detected in the chromatin accessibility analyzed by ATAC-qPCR; HLA-DR and PD-L1 displayed no altered accessibility at the gene promoters or at gene enhancers by any exposure (Figure 4A). Nor did changes occur at the promoters, the enhancers or in the gene bodies of inflammatory genes or lectin receptor genes, despite the large transcriptional induction of the inflammatory genes (Figures S4A and S4B). Many of these genes are also expressed in monocytes from which moDCs are differentiated and may therefore already have a more accessible chromatin state established. To test this, the accessibility of the response genes and moDC marker genes was examined in monocytes and in moDCs. The monocyte marker CD14 was accessible in monocytes and closed in moDCs, whereas the DC marker DC SIGN exhibited a higher accessibility in moDCs compared to the more closed configuration in monocytes (Figure 4B). The promoters of HLA-DR, PD-L1 and TNF-α also gained a higher accessibility in moDCs than in monocytes, and the promoters of IL-6, IL-1β, and CD206 had a similar high degree of accessibility in monocytes and moDCs (Figure 4C). This suggest that an open chromatin state at these genes is established during the differentiation to moDCs or even earlier. Chromatin changes are also formed by histone modifications, for instance, H3K4me3 and H3K27Ac have been associated with an active chromatin state coupled to the establishment of a trained or tolerized immune phenotype.^26^ HLA-DR and PD-L1 exhibited a trend of higher H3K27Ac levels at the promoter in cells stimulated with LPS alone (Figure 4D, upper panel), whereas no change was observed in H3K4me3 levels (Figure 4D, lower panel). No such trends in the histone modifications were observed in other groups of gene (Figures S4C and S4D), suggesting that no trained phenotype was induced at this short time span and the H3K27Ac at the promoter of HLA-DR and PD-L1 may be associated with the transcriptional activation by LPS. Taken together, the genes important for a DC response already have a hyper-accessibility chromatin structure, established in differentiation steps prior to the activation of the genes upon stimulation.

### Exposure to HZ changes the recruitment of SWI/SNF ATPase BRG1

**Figure 5:**
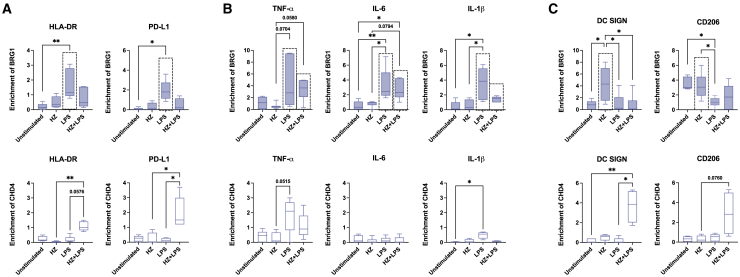
HZ changes the recruitment by LPS of BRG1 (SWI/SNF complexes) and CHD4 (NuRD) to the promoters of HLA-DR and PD-L1 (A) The enrichment of BRG1, top panel and CHD4, bottom panels, at the promoters of HLA-DR and PD-L1. The dotted box in the top panel represents the sample which had induced gene expression, *n* = 4–6. (B) The enrichment of BRG1, top panels, and CHD4, bottom panel, at the promoters of TNF-α, IL-6, and IL-1β. The dotted boxes in the top panel represent samples with induced gene expression, *n* = 4–8. (C) The enrichment of BRG1, top panels, and CHD4, bottom panel, at the promoters of DC SIGN and CD206. The dotted boxes in the top panel represent samples with maintained gene expression, *n* = 4–9. (A–C) Boxplots cover data between the 25th and the 75th percentile with median as the central line and whiskers showing min-to-max. Non-parametric Kruskal-Wallis ANOVA test, followed by Dunn’s multiple was comparison was applied to determine significant differences, n.s. = *p* > 0.05, ∗*p* < 0.05, ∗∗*p* < 0.01.

Next, we examined the association of chromatin remodeling complexes with the immune genes; SWI/SNF complexes are required for transcriptional activation of many immune genes^52^ and NuRD or EZH2 in the PCR2-polycomb complex are important for repression, in particular during the resolution phase.^37^^,^^53^^,^^54^ The ATPase BRG1/SMARCA4 in the SWI/SNF complexes was recruited to the promoters of HLA-DR and PD-L1 in LPS stimulated cells, while co-exposure with HZ and LPS had reduced levels, in agreement with the reduced levels of transcript observed (Figure 5A, upper panel). Co-exposure of HZ and LPS instead led to recruitment of the ATPase CHD4 in NuRD, but not the methyltransferase EZH2, to these promoters (Figures 5A, lower panel and S5A). The promoters of the inflammatory genes, TNF-α, IL-6, IL-1β, and CCL5, displayed higher BRG1 occupancy in cells stimulated with LPS, both in cells stimulated with LPS alone and in co-exposed cells (Figures 5B, upper panel and S5B, upper panel). However, CHD4 was only associated with these genes at low levels, except for the TNF-α promoter in LPS stimulated cells, which indicates that the repression during the resolution phase had been initiated (Figures 5B, lower panel and S5B, lower panel). The promoters of IL-23 and IL-10 had low levels of BRG1 and CHD4 at the promoters, which did not change with exposure (Figure S5C), suggesting that other factors are operating on these gene. In contrast to the other gene groups, the BRG1 levels associated with the promoters of DC SIGN and CD206 were higher in cells exposed to HZ alone, which maintained a higher transcriptional level than in LPS-stimulated cells. CHD4 associated with these promoters only in cells co-exposed to HZ and LPS (Figure 5C), possibly to allow for a reduction in transcription. This suggests that the presence of chromatin remodeling complexes is associated with differential regulation of these genes, despite no differences in chromatin accessibility.

### SWI/SNF variants operating on the genes decide gene expression of the genes

**Figure 6:**
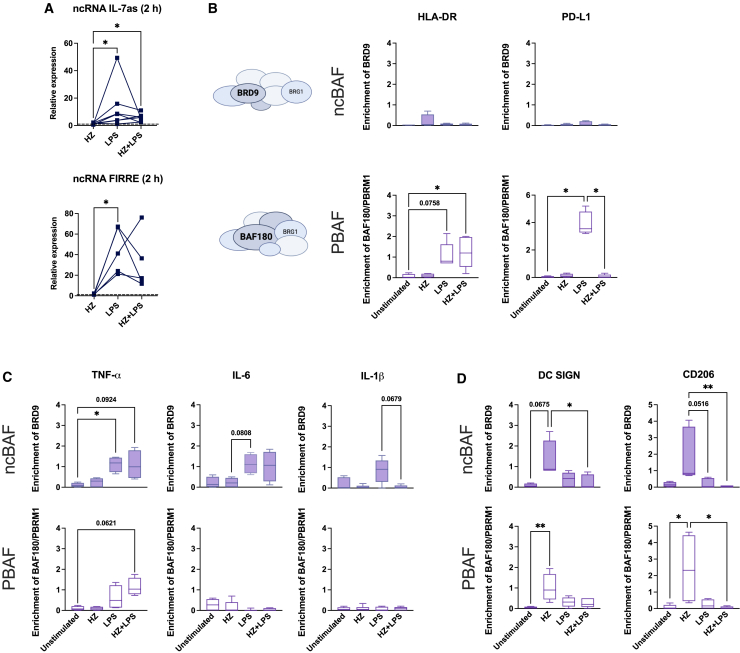
Differential recruitment of SWI/SNF complex signature proteins BRD9 and BAF180/PBRM1 at the promoters of the different gene groups (A) Relative expression of ncRNA IL-7as and ncRNA FIRRE in moDCs after 2 h exposure. The expression is related to unstimulated cells. Paired Friedman test followed by Dunn’s multiple comparison was used to determine statistical difference, n.s. = *p* > 0.05, ∗*p* < 0.05, *n* = 5–7. (B) The enrichment of BRD9 (ncBAF) and BAF180/PBRM1 (PBAF) proteins at the promoters of HLA-DR and PD-L1. Schematic complexes with the signature protein highlighted are depicted to the left of the panels, *n* = 4–6. (C) The enrichment of BRD9 (ncBAF), and BAF180/PBRM1 (PBAF) proteins at the promoters of TNF-α, IL-6 and IL-1β, *n* = 4–6. (D) The enrichment of BRD9 (ncBAF) and BAF180/PBRM1 (PBAF) proteins at the promoters of DC SIGN and CD206, *n* = 4–6. (B–D) Boxplots cover data between the 25th and the 75th percentile with median as the central line and whiskers showing min-to-max. Non-parametric Kruskal-Wallis ANOVA test, followed by Dunn’s multiple was comparison was applied to determine significant differences, n.s. = *p* > 0.05, ∗*p* < 0.05, ∗∗*p* < 0.01.

**Figure 7:**
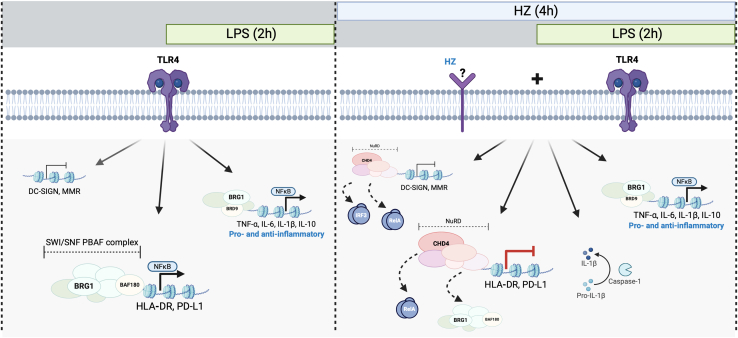
HZ dampens the LPS early acute response specifically at a subset of ISGs by exchanging the activating PBAF for the silencing NuRD complex Model of the induction of LPS through TLR4 alone to the left and co-exposure with LPS and HZ leading to the right. LPS alone induces proinflammatory genes in a RELA and BRD9-SWI/SNF-dependent manner and HLA-DR and PD-L1 in a RELA and BAF180-SWI/SNF-dependent manner. Co-exposure of LPS and HZ leads to NuRD replacing BAF180-SWI/SNF and RELA at the HLA-DR and PD-L1 promoters, dampening the transcriptional response.

To investigate the specificity of factor binding to the different gene types, we determined the expression of ncRNAs regulating the binding of BRG1-SWI/SNF to immune genes. The recruitment of SWI/SNF complexes to immune genes in human cells is affected by two LPS-regulated lncRNAs, IL-7-as and FIRRE, in opposite ways; IL-7-as enhances, and FIRRE reduces the association.^55^^,^^56^ Both RNA levels were induced by LPS stimulation and co-exposure to LPS and HZ for 2 h compared to cells exposed to HZ alone and unstimulated cells (Figure 6A), which indicates that the expression of these RNAs does not explain the differential recruitment of factors to the promoters in LPS and co-exposed cells. Instead, we examined whether the promoters associated with different SWI/SNF complexes and used BRD9 as a marker for the non-canonical SWI/SNF (ncBAF complex) and BAF180/PBRM1 for PBAF. BAF180 was preferentially recruited to the HLA-DR and PD-L1 promoters upon LPS stimulation for 2 h, and the binding was prevented by HZ exposure at the PD-L1 promoter (Figure 6B). This demonstrates that these genes worked with the PBAF constellation to activate transcription and since co-exposure also recruited the NuRD complex, we hypothesize that these two complexes function together to decrease transcription when required. The promoters of inflammatory genes TNF-α, IL-6 and IL-1β showed a preference for the BRD9 subunit, with higher promoter association upon LPS stimulation and co-exposure (Figure 6C). HZ-exposure to DC SIGN and CD206 led to both BRD9 and BAF180 associating at their promoters (Figure 6D), but both subunits were lost in the co-exposed cells together with BRG1 and replaced by CHD4. In conclusion, the specificity in the transcriptional response to HZ was correlates with the recruitment of specific SWI/SNF complexes, with different features, to the various promoters (as depicted in Figure 7).

## Discussion

Malaria co-infection with bacteria accelerates both the malaria progression and the bacterial infection.^57^^,^^58^ Infections with malaria in endemic areas are known to reduce the immune response to further infections, but may also induce a trained phenotype with heightened response to subsequent infections. However, the cellular mechanism in response to simultaneous infections are poorly understood and contradictory results have been reported.^2^ To address the question about the effects of a co-infection with malaria and bacteria, we used the malaria product HZ to study its effect on the immediate immune response in malaria naive individuals. Malaria-naive individuals respond to malaria infections differently to malaria-experienced individuals, inducing pro-inflammatory and interferon-stimulated genes (ISG) stronger and also differently.^6^ We show that HZ only had small direct effects on the expression of immune genes in the early acute phase, instead it modulated the transcriptional response to LPS, a bacterial stimulus. HZ exposure of moDCs did not induce an early pro-inflammatory response as we could not detect secretion of inflammatory cytokines or chemokines. iRBC lysate on blood-derived DCs^14^ also shows a lack of a pro-inflammatory response and the RNA-seq performed on the cells after 3 h of exposure shows that the main group of genes affected early is related to lipid synthesis and only a minor part of the immune genes is upregulated; in particular in the TLR2/MYDD88/IRAK1 pathway.^14^ Instead for a direct transcriptional effect on immune associated genes, we detected that HZ exposure compromised the early LPS-induced transcriptional activation of HLA-DR, PD-L1 and CXCL10, by altering the recruitment of transcription factors and chromatin remodeling complexes at the promoters. We show that activating SWI/SNF complexes were replaced by the silencing NuRD complex during co-infection on these genes as well as DC SIGN and CD206 (Figure 7), but not on inflammatory genes. In addition, it has been shown that HZ exposure only partially mature moDCs^19^ and this can be due to a maintained gene expression of the immaturity markers DC SIGN and CD206. Taken together, HZ exposure leads to a dampening or skewing of subsequent immune responses, including T cell activation, without affecting the inflammatory response to bacterial infections transiently in the early acute phase.

The transcriptional response is regulated by activation of transcription factors and we show that in addition to HZ and LPS, which activate the NF-κB factor RELA,^7^^,^^8^^,^^24^^,^^32^^,^^33^ HZ activated IRF3 after 2 h. The receptor for HZ or HZ-associated molecules is not known and several receptors have been suggested; the urine crystal receptor CLEC12,^49^ the TLR/CD11b^24^ or any other form of endocytosis.^5^ Not only the heme crystal but also associated molecules have been attributed to the response,^3^^,^^7^^,^^24^ including lipid products which can act on specific receptors.^10^ Furthermore, the effect of HZ could also be attributed to HZ-transported molecules and newly produced HZ molecules.^9^^,^^10^ However, the stimulatory effect of β-hematin, the pure crystal, on DC maturation genes was shown to be similar to that of opsonized HZ.^19^ The innate immune response in human cells is slightly different from that in mouse cells, using different signaling effectors and scaffolding proteins.^40^^,^^41^^,^^42^^,^^50^ We found that HZ activated IRF3, which is most likely through endosome internalization. IRF3 is involved in the acute response to viral infections through intracellular receptors in human cells^50^; in pDCs and DC cell lines, IRF3 induction depends on RIG I/MAVS and possible cGAS/STING, whereas IRF5 is induced upon TLR activation, and these transcription factors activate separate repertoires of targets, also in a time-dependent manner.^51^ In addition, IRF3 is expressed at low levels in human macrophages, as is the signaling adaptors TRAF and TBK1.^41^^,^^50^ Analysis of the published RNA-seq dataset of the iRBC from mDCs^14^ shows a more prone IRF5 signature than an IRF3 response. Nevertheless, genes such as IL1-RA, identified as sole IRF3 targets,^50^ had IRF3 associated with the promoter in HZ exposed cells, indicating that HZ or possibly iRBC activates a virus-like response in human moDCs through intracellular receptors.

Although both NF-κB and IRF3 were activated by HZ-exposure, the factors were differentially recruited to the various genes; activated IRF3 was recruited to the promoters of DC SIGN and CD206 and not to other IRF3 targets, such as CCL5. Furthermore, RELA was recruited to by LPS stimulation on inflammatory genes, but only by LPS alone to the promoters of HLA-DR and PD-L1. The specific response of genes depends on many factors, such as their promoter and enhancer architecture as well as on their chromatin state. HLA-DR, PD-L1 and CXCL10 are ISG^59^^,^^60^^,^^61^ with promoters harboring interferon-stimulated response elements (ISRE) that are specifically responsive to IFNγ through the activation of the JAK-STAT pathway by the STAT1 and IRFs.^62^^,^^63^ These gene promoters also harbor NF-κB binding sites and are regulated by TLR signaling through early activation of the NF-κB transcription factors RELA in response to pathogens.^47^^,^^48^^,^^64^ We performed a computational comparison between the ISGs affected and inflammatory genes by ConTtra v3, update 2017, which showed that the ISGs contained more STAT1 and IRF-sites, while the inflammatory genes had more NF-κB elements. This analysis showed that while LPS stimulation recruited RELA to both ISGs and inflammatory genes, co-exposure with HZ reduced only the recruitment of RELA to specific ISGs. This varied response of different genes may be caused by different transcription factors activated at different times^41^ but also attributed to different signaling “codons”.^47^ Single cell RNA transcription analyses have shown that although genes respond to the same transcription factors, they are activated at different time points and at different amplitudes.^41^^,^^47^^,^^60^^,^^64^ The response is depending on the type and quality of signals triggered by the stimulus: the types of signal secreted by neighboring cells, autoregulatory signals and the chromatin states in individual target cells.^47^^,^^48^^,^^61^^,^^62^ These variations in the timing and amplitude of signals most likely contribute to the specific gene expression response of different type of gene with different gene architecture.

A further point is cross-talk between signaling pathways, which also results in variations of the individual gene response. The response of HLA-DR and PD-L1 in human cells depends on factors such as IRF1, which is induced by LPS-TLR4 in human macrophages and is associated, together with the early induction of IFNB1, to an autocrine loop inducing interferon inducible genes.^41^ We detected an induction of IRF1 by LPS and HZ co-exposure, but only after 6 h compared to 2 h in cells exposed only to LPS, and at a lower level. Several layers of cross-talk exist between the TLR and IFN-pathways and IRF1 together with STAT1 is associated with priming of pro-inflammatory genes for a more pronounced transcriptional response in human macrophages and monocytes^65^ and STAT1 and IRF1 can directly activate genes such as PD-L1 in human DCs.^66^^,^^67^ Analyses of the published RNA-seq in stimulated mDCs^14^ show an induction of IRF1, but not of IRF5 or IRF3, after LPS stimulation. This intricate cross-talk between pathways may allow for a later LPS/TLR4-dependent gene expression of HLA-DR and PD-L1 in co-exposed cells, resulting in a surface expression similar to that of LPS alone after 24 h.

Accessible chromatin and active histone modifications are often correlated with active transcription, but we could only detect minor changes in chromatin accessibility or in the levels of histone H3K4me3 and histone H3K27Ac at the promoters of the genes upon treatments compared to unstimulated cells after 2 h. Exposure to either iRBC or HZ for of at least 24 h induces a trained phenotype with increased enrichment of H3K4me3 at specific genes in monocytes,^29^^,^^30^ which suggests that short-term exposure requires different mechanisms to induce transcription. The accessibility of many gene promoters is also established during differentiation in processes involving changes in histone modifications.^68^ Differentiation of monocytes to moDCs includes loss of H3K4me3 at the promoter of the monocyte marker CD14 gene and a gain at the DC SIGN gene.^69^ Furthermore, specific chromatin remodeling complexes, among them different SWI/SNF complexes, are involved at different differentiation steps in hematopoiesis.^70^ In T cell differentiation, chromatin priming and the maintenance of a poised state at effector genes are associated with specific lineage transcription factors and SWI/SNF complexes.^38^^,^^71^ We investigated the accessibility during moDCs differentiation of the genes involved in DC maturation and they had either gained an open configuration during the differentiation to moDCs or already had an open configuration established in an even earlier step. Our results showed that maturation of DCs does not require further alteration of the accessibility at the promoters in the early acute phase and the transcriptional induction of DC genes depends on the activation and binding of transcription factors and the association with chromatin remodeling complexes.

Many immune genes associate with SWI/SNF complexes and they are often required for transcriptional activation upon immune stimulation.^37^^,^^52^^,^^59^ In the resolution phase, when the inflammation should be dampened to prevent tissue damage, activating transcription factors are replaced with others, for instance RELA is replaced by a homodimer of p50-p50.^72^ Resolution also involves either the NuRD complex that antagonizes SWI/SNF activation^34^^,^^60^ or the EZH2-PCR2 that silences genes by forming polycomb heterochromatin.^53^^,^^54^ We observed that the promoters of several of the investigated genes, including HLA-DR and PD-L1, associated with SWI/SNF ATPase BRG1 when activated by LPS, and that BRG1 was replaced by the NuRD ATPase CHD4 when transcription was reduced following HZ and LPS co-exposure. We did not detect EZH2, which confers polycomb silencing in cancer cells,^53^^,^^54^^,^^73^ on the promoters. NuRD is involved in silencing of ISG genes, including PD-L1 and CXCL10, in epithelial cell upon long-term infection.^74^ BRG1 and CHD4 are both involved in nucleosome shifting locally of the +1 or −1 nucleosome at the TSS,^75^^,^^76^ which changes the binding of RNA pol II; in embryonic stem cells BRG1 increases the RNA pol II binding^75^^,^^76^ whereas the NuRD excludes RNA pol II.^77^^,^^78^ At highly expressed genes not having bivalent histone marks, the SWI/SNF and NuRD complexes are involved in transcriptional changes by regulating RNA pol II occupancy at the gene promoter and in the gene body.^78^ Because of the weak shift in accessibility upon activation, we propose that in the early acute phase, a subgroup of ISGs is induced by activated RELA together with the SWI/SNF complex PBAF and resolution factors recruit the NuRD complexes. This leads to an altered RNA pol II kinetics rather than causing large changes in chromatin configuration at genes already in an open, poised state. In this context, HZ exposure establishes a different balance of the chromatin complexes at the HLA-DR and PD-L1 genes as well as on the C-lectin genes, which results in a compromised gene expression in response to concomitant LPS stimulation (as depicted in Figure 7).

Different SWI/SNF constellations operate on various regulatory elements, regulating cell type specific expression^37^^,^^79^^,^^80^ and occupying enhancers of different genes in mouse bone-marrow derived macrophages (BMDM).^38^ We show that the ISG gene promoters of HLA-DR and PD-L1 were associated with BAF180-PBAF with upon LPS stimulation, which is replaced by the NuRD complex in co-exposed cells and reduced transcription. PBAF association to promoters often leads to repression, such as repression of genes in T-regs and IFNγ response genes.^36^^,^^37^^,^^81^^,^^82^^,^^83^ In contrast to the PBAF complex operating on HLA-DR and PD-L10, we found that a number of the inflammatory genes associated with the BRD9-ncBAF upon LPS stimulation, and this complex was not antagonized by NuRD upon co-exposure with HZ. Analyses of the ChIP-seq and expression datasets from stimulated mouse BMDM^38^ revealed that PBAF occupied regions in the vicinity of the PD-L1 and CXCL10 promoter and that the ncBAF was associated with inflammatory genes after 4 h of stimulation. We propose that PBAF is preferentially antagonized by the NuRD complex, which may render PBAF more repressive. Co-exposure to HZ and LPS did not markedly change the secretion of pro-inflammatory cytokines apart from IL-1β, which was only secreted from co-exposed cells after 24 h of stimulation and not from cells exposed to HZ and LPS alone. The induction of IL-1β requires both the transcriptional activation of the IL-1β gene and other inflammasome genes as well as the activation and assembly of the inflammasome.^84^ HZ did not induce the transcription of IL-1β in moDCs but the activation of the inflammasome has been attributed to HZ by activating the STAT pathway through LYN/SYK kinases^42^ or to induce the production of ROS.^33^^,^^42^ Co-infections may therefore contribute to an excessive pro-inflammatory response. RNA-seq of the response after 24 h in mouse liver following HZ (with plasmodium DNA) exposure resulted in an innate immune IFNγ signature response with induced IL1β transcripts.^85^ This response is linked to resident macrophages and NK-cells and the difference in the responses in mouse liver and human moDCs may be cell type specific. The innate response to HZ in mouse liver correlates with a more transient protection to decrease the parasite load, and we suggest that HZ have an immune modulative effect on DCs during the early blood stage to maintain DCs in a partially immature state and dampen the response. Equally important is the early modulation of the innate response, in which HZ-exposure pre-disposes some genes for the response to other pathogens. We propose that the gene architecture determines what type of SWI/SNF complex that associates with the promoter, which in turn determines subsequent regulatory events. In particular, PBAF and NuRD are exchanged at a subset of INFγ regulated promoters in co-exposed cells. The exchange of complexes did not alter the chromatin state, and is more likely to change the RNA pol II kinetics. This may fine-tune a homeostatic response to avoid random maturation and also modulate the response toward a skewed immune response by subsequent or simultaneous bacterial infections and lead to a more severe disease during co-infections.

### Limitations of the study

This study is based on moDCs differentiated from monocytes from healthy blood donors in Sweden. moDCs only make up a fraction of the DCs in blood or tissues, however, they mimic mDCs/cDCs in response even though the origin may be different. We have used moDCs from healthy adult Swedish donors of both sexes, with no recent infection history, no HIV, no prior malaria, and no congenital defects. Thus, the blood samples serve as a source of a statistically normal population of non-activated healthy monocytes, to avoid tolerance or trained phenotypes, or other confounding factors. Nevertheless, the healthy controls are genetically diverse and have different immunological reactions. We have therefore repeated the experiment in several samples as specified in the Figure Legends, but the number is still low when regarding natural populations. Because of the low number, we have only studied general responses in malaria-naive populations, and not investigated any sex-differences. Despite the low number of samples, we could see trends and the results may form the basis for further larger studies, in particular from controlled cohorts, in which sex-differences also could be a factor. In addition, the large genetic diversity in the subjects prompted us to perform a targeted investigation, examining the expression of genes and gene products known to react in the early response of DCs instead of global examinations.

We used opsonized HZ preparation to mimic the HZ released from the erythrocytes. Different preparations of HZ have been established which vary in composition and many cargo components from the infected erythrocyte and blood may trigger an immune response. Our HZ preparation has been used in several publications and is thoroughly tested, for example for LPS contaminations.^10^ However, the variation of cargoes of different HZ preparations may give slightly different results.

## Resource availability

### Lead contact

Further information and any related requests should be directed to and will be fulfilled by the lead contact, Ann-Kristin Östlund Farrants (anki.ostlund@su.se).

### Materials availability

This study did not generate new unique reagents.

### Data and code availability

All data reported in this paper will be shared by the lead contact upon request. This paper does not report original code.

## Acknowledgments

This research was funded by The Swedish Cancer Society (19 0453 Pj and 22 2310 Pj to A.-K.Ö.F. and 20 1117 Pj and 23 2985 Pj to E.S.-E.), The 10.13039/501100004359Swedish Research Council (2020-01839 and 2023-02616 to E.S.-E.), 10.13039/501100002805Carl Trygger Foundation (CTS 22:1988 to A.-K.Ö.F) and 10.13039/501100009244Stockholm University.

## Author contributions

A.-K.Ö.F., E.S.-E., and M.T.-B. designed the study. G.L., K.T., A.S., M.P., J.Q., A.-K.Ö.F., and I.B. performed lab work. G.L., K.T., A.S., J.Q., I.B., M.P., E.S.-E., and A.-K.Ö.F. analyzed and/or finalized the data. G.L. performed statistical analyses. A.-K.Ö.F., G.L., and E.S.-E. wrote the paper with input from A.S., K.T., and M.P.

## Declaration of interests

The authors declare no competing interests.

## STAR★Methods

### Key resources table

**Table:** 

REAGENT or RESOURCE	SOURCE	IDENTIFIER
**Antibodies**		

Anti-RELA/NFκB p65	Abcam	Abcam Cat# ab7970; RRID: AB_306184
Anti-IRF3 (ChIP)	Abcam	Abcam Cat# ab76409; RRID: AB_1523835
Anti-CHD4	Abcam	Abcam Cat# ab240640; RRID: AB_2941814
Anti-BRG1	Östlund Farrants et al.^86^	Reference [86]
Anti-histone H3	Abcam	Abcam Cat# ab1791; RRID: AB_302613
Anti-histone H3K27Ac	Abcam	Cat# ab4729; RRID: AB_2118291
Anti-histone H3K4me3	Abcam	Abcam Cat# ab8580; RRID: AB_306649
Anti-BAF180/PBRM1	Bethyl Laboratories Inc	Cat# A700-019; RRID: AB_2891820
Anti-BRD9	Bethyl Laboratories Inc	Cat# A303-781A; RRID: AB_11218396
IgG	Abcam	Cat# ab171870; RRID: AB_2687657
Anti-phospho-IRF3 (Ser-396)	Cell Signaling Technology	Cat# 53539; RRID: AB_2799439
Anti-IRF3 (immunoblot)	Cell Signaling Technology	Cat# 11904; RRID: AB_2722521
anti-Caspase I	Abcam	Cat# ab207802; RRID: AB_2889889
Anti-Lamin B1	Abcam	Cat# ab65986; RRID: AB_1140888
Anti-tubulin	Abcam	Cat# ab6046; RRID: AB_2210370
Goat Anti-Rabbit IgG H&L (HRP)	Abcam	Cat# ab6721; RRID: AB_955447
Anti-human-CD11c APC (clone 3.9)	BioLegend	Cat# 301614; RRID: AB_493023
Anti-MMR PerCP-Cy5.5 (clone 15-2)	BioLegend	Cat# 141715; RRID: AB_2561991
Anti-human-TLR4 PE (clone HTA125)	BioLegend	Cat# 312805; RRID: AB_314954
Anti-human -CLEC12A FITC (clone 50C1)	BioLegend	Cat# 353608; RRID: AB_2563623
Anti-human-CD83 PeCy-7 (clone HB15e)	BioLegend	Cat# 305325; RRID: AB_256177
Anti-human-HLA-DR PerCP (clone L243)	BioLegend	Cat# 980420; RRID: AB_2922651
Anti-human-PD-L1 PE (clone 29E.2A3)	BioLegend	Cat# 329708; RRID: AB_940360
Anti-human-DC SIGN BV421 (clone DCN46)	BD Biosciences	Cat# 566278; RRID: AB_2739652
Anti-human-CD86 FITC (clone FUN-1)	BD Biosciences	Cat# 557343; RRID: AB_396651

**Chemicals, peptides, and recombinant proteins**		

Fixable Viability Stain 780	BD Biosciences	Cat# 565388
Penicillin-Streptomycin, Hyclone™	Cytiva	Cat# SV30010
L-glutamine, Hyclone™	Cytiva	Cat# HYCLSH30034.0
HEPES buffer solution, Hyclone™	Cytiva	Cat# HYCLSH30237.01
RPMI-1640, Hyclone™	Cytiva	Cat# SH30027.FS
Human AB serum	Sigma Aldrich	Cat# H4522
Sodium pyruvate, Gibco™	Thermo Fisher Scientific	Cat# 11360070
2-Mercaptoethanol, Gibco™	Thermo Fisher Scientific	Cat# 21985023
Human IL-4 Recombinant Protein, PeproTech®	Thermo Fisher Scientific	Cat# 200-04
Human GM-CSF Recombinant Protein, PeproTech®	Thermo Fisher Scientific	Cat# 300-03
LPS from *E. coli* O26:B6	Sigma Aldrich	Cat#: L2654
Mannitol	Sigma Aldrich	Cat# M9647
Chloroform	BDH, VWR Life Sciences	Cat# 83627.400
Isoamyl alcohol, ultrapure	VWR Life Sciences	Cat# 97065-028
2-Propanol	VWR Life Sciences	Cat# 97064-700
Formaldehyde, 37%, Mol. Biology grade	Merck, Sigma Aldrich	Cat# 47608-250 ml
Phenol, 89% solution	Merck, Sigma Aldrich	Cat# P9346

**Critical commercial assays**		

Ficoll-Paque™ PLUS	Cytiva	Cat# 17-1440-03
EasySep™ human monocyte kit	STEMCELL Technologies	Cat# 19059
Percoll	Sigma Aldrich	Cat# P1644
Tri Reagents	ThermoFisher Scientific	Cat# AM9738
Superscript VILO cDNA Synthesis kit	ThermoFisher Scientific	Cat# 11754050
Tn5 transposase (Illumina Tagment DNA TDE1 Enzyme and Buffer Kits)	Illumina	N/A
MinElute PCR purification kit	Qiagen	Cat# 28004
Clarity -ECL-Western substates	BioRad	Cat# 1705060
Kapa SYBR FAST qPCR mix	Roche/KAPA Biosystems, Sigma Aldrich	Cat# KK4618
CBA human Inflammatory Cytokine Kit	BD Biosciences	Cat# 551811
CBA human Chemokine kit	BD Biosciences	Cat# 552990
Elisa FLEX: human TNF-α (ALP)	Mabtech	Cat# 3512-1A-6
Elisa FLEX: human IL-6 (ALP)	Mabtech	Cat# 3460-1A-6
Elisa FLEX: human IL-23 (ALP)	Mabtech	Cat# 3457-1A-6
Elisa FLEX: human IL-10 (ALP)	Mabtech	Cat# 3430-1A-6
Elisa FLEX: human IL-1β (ALP)	Mabtech	Cat# 3416-1A-6
Human IL-1ra/IL-1F3 DuoSet ELISA	Biotechne, R&D systems	Cat# DY280

**Experimental models: Organisms/strains**		

Human buffy coats	Stockholm Blood Centre	N/A

**Oligonucleotides**		

Presented in Table S1		

**Software and algorithms**		

SoftMax Pro 5.2 rev C software	Molecular Devices Corp	
ImageLab (ChemiDoc™ Imaging system)	BioRad	
FlowJo software v10.10	BD Biosciences	
Graph Pad Prism software v10.4.1	GraphPad software	
BioRender software (used for Figures 1A and 7, Graphical abstract)	BioRender	https://biorender.com;
ConTra v3, update 2017	Inflammation Research Centre, Ghent University	Łukasz Kreft et al.^87^

**Other**		

Protein A/G magnetic beads	ThermoFisher Scientific	Cat# LSKMAGAG
PVDF immobilon membrane	Milipore/Sigma Aldrich	Cat# HVLP04700

### Experimental model and study participant details

Peripheral blood cells were isolated from buffy coats retrieved from anonymous, healthy blood donors (Stockholm Blood Centre) of both sexes, with no recent infection history, no HIV, no prior malaria, and no congenital defects. the blood samples serve as a source of a statistically normal population of non-activated healthy monocytes, to avoid tolerance or trained phenotypes. The identity cannot be traced back; thus, the project does not require approval from the Swedish ethical review authority.

#### Primary cell isolation and stimulations

Peripheral blood mononuclear cells (PBMC) were isolated from buffy coats using Ficoll-Paque™ PLUS (Cytiva) gradient centrifugation. Monocytes were enriched from PBMC by negative selection using EasySep™ human monocyte kit (STEMCELL Technologies) according to the manufacturer’s instructions. Monocytes were resuspended at 1 × 10^6^ cells/mL and were seeded in 96-well plates (with 200 μl/well) or 6-well plates (with 2 ml/well) using RPMI-1640 culture medium which was supplemented with 20 mM HEPES, 2 mM L-glutamine, 100 U/mL penicillin, 100 μg/ml streptomycin (all from Cytiva), 5% human AB serum (Sigma-Aldrich), 50 μM 2-mercaptoethanol, 2% sodium pyruvate (both from Gibco, Thermo Fisher Scientific), 35 ng/ml IL-4 and 50 ng/ml GM-CSF (both from PeproTech, Thermo Fisher Scientific). On day 3, half of the medium volume was replaced with the fresh medium containing IL-4 and GM-CSF. On day 5, cells were pre-exposed to HZ (5 μl for 96-well plates and 50 μl for 6-well plates) and the plates were centrifuged at 700 r.p.m. for 20 s in order to initiate the contact with Hz. After 2 h, 50 ng/ml of LPS from *Escherichia coli* O26:B6 (Sigma-Aldrich) was added to appropriate wells for additional 2 h or 24 h. Cells assigned to HZ treatment alone were exposed to HZ at the same time as was added LPS. Cells kept in complete culture medium served as the control. Supernatants were collected and stored at -20°C until further analysis, while cells were collected either for western blot, chromatin immunoprecipitation (ChIP), mRNA expression analysis, ATAC-qPCR or flow cytometry. Throughout the experiment, cells were incubated at 37°C with 5% CO_2_.

### Method details

#### Natural HZ preparation

Natural HZ was prepared as previously described.^9^^,^^10^^,^^19^ Briefly, 12 h after schizogony of synchronized FCR3 parasite cultures, the supernatant was collected. A 10%/40% interphase of a 6% mannitol-containing Percoll (Sigma Aldrich) gradient was used to collect natural HZ, which was then extensively washed with 10 mM phosphate buffer followed by washing with PBS. HZ was opsonized with equal volume of human AB serum (Sigma Aldrich) for 30 min at 37°C and then it was quantified according to its haem content. HZ preparation with 4 nmol/μl haem content was used in the study. Prior to use, HZ was resuspended by passage through a 0.4 mm needle fitted to a 1 ml syringe. The type of preparation was tested for LPS contaminations a referred to in Skorokhod et al.^10^

#### Immunoblotting

MoDC were harvested in 4×Lameli buffer, separated on a 12% PAGE and transferred to a PVDF Immobilon membrane (Milipore, Sigma Aldrich). The membrane was blocked in 5% milk and incubated separately with primary antibodies: rabbit monoclonal anti-Phospho-IRF3 (Ser-396) (D601M, Cell Signaling), rabbit monoclonal anti-IRF3 (D6I4C, Cell Signaling), rabbit polyclonal anti-Lamin B1 (ab65986, Abcam), rabit monoclonal anti-Caspase-1 (ab207802, Abcam), rabbit polyclonal anti-beta Tubulin (ab6046, Abcam) over night. Immunoblots were developed using Clarify ECL Western Substrate (BioRad) after incubated with goat-anti rabbit IgG-HRP conjugated secondary antibody (ab8721, Abcam) . The quantification of protein signals was performed by ImageLab™ (ChemiDoc™ Imaging system, BioRad) software by comparing the signals from each target proteins to that of loading control proteins as specified in the Figure Legends.

#### Chromatin immunoprecipitation

Chromatin from different treatment groups was cross-linked using 1% formaldehyde (Merck, Sigma-Aldrich), sonicated for 45 minutes 30 minutes on-off cycles on ice. Chromatin from 1 × 10^6^ cells was incubated with antibodies against histone H3 (ab1791), histone H3K27Ac (ab4729), histone H3K4me3 (ab8580), RELA/NFκB p65 (ab7970), IRF3 (ab76409), CHD4 (ab240640), BRD9 (ab314091), BAF180/PBRM1 (ab243876), IgG (ab171870) (all from Abcam), or BRG1 antibody.^86^ Protein A/G magnetic beads (Thermo Fisher Scientific™) were used to precipitate target chromatin fragments and the DNA extracted using Phenol:Chloroform:Isoamyl alcohol (PCI) (25:24:1) at pH 7.4 (from Sigma Aldrich and VWR Life Sciences) . The target DNA was amplified by qPCR using KAPA SYBR FAST qPCR Master mix (Kappa Biosystems Inc). The results are presented as percentage of input, with the IgG control subtracted from the Ct values obtained for samples. Analysis of predicted transcription factor binding sites was performed using ConTra v3, update 2017.^87^

Primers are listed in the Table S1. For the sonication test, sonicated chromatin from 0.5 × 10^6^ cells was extracted with PCI (25:24:1) at pH 7.4 and precipitated.

#### RNA extraction and real-time quantitative polymerase chain reaction

Total RNA was extracted using TRI reagent (Thermo Fisher Scientific™), and reverse transcription of RNA was performed using SuperScript VILO cDNA synthesis kit (Thermo Fisher Scientific™), according to the manufacturers’ instructions. qPCR was performed using KAPA SYBR FAST qPCR Master mix (Roche/KAPA Biosystems, Sigma Aldrich). mRNA expression levels were calculated using 2^-ΔΔCt^ method and PP1A was used for normalization. Primer pair list can be found in the Table S1.

#### ATAC-qPCR

Cells were collected and tagged with Tn5 transposase (Tagment DNA TDE1 Enzyme and Buffer Kits, Illumina) according to the manufacturer’s instructions. DNA was purified using MinElute PCR purification kit (Qiagen) and it was stored at -20°C until further analysis by qPCR. Tagmentation-specific forward primer and gene-specific reverse primer was used for amplification. IL-2 was used for normalization. Primer pair list can be found in the Table S1.

#### Flow cytometry

Following cell collection on day 5 or day 6, moDC were stained with alternating combination of antibodies: CD11c APC (clone 3.9), MMR PerCP-Cy5.5 (clone 15-2), TLR4 PE (clone HTA125), CLEC12A FITC (clone 50C1), CD83 PeCy-7 (clone HB15e), HLA-DR PerCP (clone L243), PD-L1 PE (clone 29E.2A3) (all from BioLegend), DC SIGN BV421 (clone DCN46) and CD86 FITC (clone FUN-1) (both from BD Biosciences). A fixable viability stain 780 (BD Biosciences) was used to exclude non-viable cells. Samples were acquired using FACSVerse flow cytometer (BD Biosciences) and FlowJo Software v10.10 (TreeStar) was used to analyse the data.

#### Cytometric bead array (CBA)

Soluble levels of TNF-α, IL-10, IL-6, IL-1β, and IL-12 cytokines were measured in the cell culture supernatant after 2 h of exposure using CBA human Inflammatory cytokine kit (BD Biosciences), while chemokines CXCL8/IL-8, CCL5/RANTES, CXCL9/MIG, CCL2/MCP-1 and CXCL10/IP-10 were quantified using CBA human chemokine kit (BD Biosciences), according to the instructions from the manufacturer. Samples were acquired using FACSVerse flow cytometer and FlowJo Software v10.10 was used for data analyses.

#### Enzyme-linked immunosorbent assay

Soluble levels of TNF-α, IL-6, IL-1β and IL-10 (MabTech AB) in the cell culture supernatant were measured in separate ELISAs at 24 hours and IL-23 (MabTech AB) and IL-RA (Biotechne, R&D systems), were measured after 2 h and 24 h in the exposed samples according to the instructions from the manufacturer. The results were analysed using SoftMax Pro 5.2 rev C (Molecular Devices Corp.).

### Quantification and statistical analysis

Statistical analysis was carried out using GraphPad Prism 10 (GraphPad Prism Inc.). Non-parametric Friedman ANOVA test or non-parametric Kruskal-Wallis ANOVA test, followed by Dunn’s Multiple Comparison Test was applied to determine the differences between multiple treatment conditions. Non-parametric Wilcoxon matched-pairs signed rank test or non-parametric Mann-Whitney test was used to determine the differences between two treatment conditions. Statistical differences were considered significant if p-values were <0.05. The number of donors (n) in each experiment is given in the Figure Legends for each graph, as well as which statistical test used.

The quantification of protein signals in the immunoblots was performed by ImageLab™ (ChemiDoc™ Imaging system, BioRad) software by comparing the signals from each target proteins to that of loading control proteins specified in the Figures legends. The number of samples (n) is specified in the Figure Legends.

## References

[1] Milner D.A. (2018). Malaria pathogenesis. Cold Spring Harb. Perspect. Med..

[2] Harding C.L., Villarino N.F., Valente E., Schwarzer E., Schmidt N.W. (2020). Plasmodium impairs antibacterial innate immunity to systemic infections in part through hemozoin-bound bioactive molecules. Front. Cell. Infect. Microbiol..

[3] Pham T.T., Lamb T.J., Deroost K., Opdenakker G., Van den Steen P.E. (2021). Hemozoin in malarial complications: more questions than answers. Trends Parasitol..

[4] Aldridge J.R., Vogel I.A. (2014). Macrophage biology and their activation by protozoan-derived glycosylphosphatidylinositol anchors and hemozoin. J. Parasitol..

[5] Olivier M., Van Den Ham K., Shio M.T., Kassa F.A., Fougeray S. (2014). Malarial pigment hemozoin and the innate inflammatory response. Front. Immunol..

[6] Tran T.M., Jones M.B., Ongoiba A., Bijker E.M., Schats R., Venepally P., Skinner J., Doumbo S., Quinten E., Visser L.G. (2016). Transcriptomic evidence for modulation of host inflammatory responses during febrile Plasmodium falciparum malaria. Sci. Rep..

[7] Skorokhod O.A., Alessio M., Mordmüller B., Arese P., Schwarzer E. (2004). Hemozoin (malarial pigment) inhibits differentiation and maturation of human monocyte-derived dendritic cells: a peroxisome proliferator-activated receptor-γ-mediated effect. J. Immunol..

[8] Tembo D., Harawa V., Tran T.C., Afran L., Molyneux M.E., Taylor T.E., Seydel K.B., Nyirenda T., Russell D.G., Mandala W. (2023). The ability of Interleukin-10 to negate haemozoin-related pro-inflammatory effects has the potential to restore impaired macrophage function associated with malaria infection. Malar. J..

[9] Skorokhod O., Barrera V., Mandili G., Costanza F., Valente E., Ulliers D., Schwarzer E. (2021). Malaria pigment hemozoin impairs GM-CSF receptor expression and function by 4-hydroxynonenal. Antioxidants.

[10] Skorokhod O., Barrera V., Valente E., Ulliers D., Uchida K., Schwarzer E. (2025). Malarial pigment induced lipoperoxidation, inhibited motility and decreased CCR2 and TNFR1/2 expression on human monocytes. Redox Exp. Med..

[11] Pinzon-Charry A., Woodberry T., Kienzle V., McPhun V., Minigo G., Lampah D.A., Kenangalem E., Engwerda C., López J.A., Anstey N.M., Good M.F. (2013). Apoptosis and dysfunction of blood dendritic cells in patients with falciparum and vivax malaria. J. Exp. Med..

[12] Wu S., Nie Q., Tan S., Liao G., Lv Y., Lv C., Chen G., Liu S. (2023). The immunity modulation of transforming growth factor-β in malaria and other pathological process. Int. Immunopharmacol..

[13] Couper K.N., Barnes T., Hafalla J.C.R., Combes V., Ryffel B., Secher T., Grau G.E., Riley E.M., de Souza J.B. (2010). Parasite-derived plasma microparticles contribute significantly to malaria infection-induced inflammation through potent macrophage stimulation. PLoS Pathog..

[14] Götz A., Tang M.S., Ty M.C., Arama C., Ongoiba A., Doumtabe D., Traore B., Crompton P.D., Loke P., Rodriguez A. (2017). Atypical activation of dendritic cells by Plasmodium falciparum. Proc. Natl. Acad. Sci. USA.

[15] Giusti P., Urban B.C., Frascaroli G., Albrecht L., Tinti A., Troye-Blomberg M., Varani S. (2011). Plasmodium falciparum-infected erythrocytes and beta-hematin induce partial maturation of human dendritic cells and increase their migratory ability in response to lymphoid chemokines. Infect. Immun..

[16] Götz A., Ty M.C., Rodriguez A. (2019). Oxidative stress enhances dendritic cell responses to Plasmodium falciparum. Immunohorizons.

[17] Ty M.C., Zuniga M., Götz A., Kayal S., Sahu P.K., Mohanty A., Mohanty S., Wassmer S.C., Rodriguez A. (2019). Malaria inflammation by xanthine oxidase-produced reactive oxygen species. EMBO Mol. Med..

[18] Mandala W.L., Msefula C.L., Gondwe E.N., Drayson M.T., Molyneux M.E., Maclennan C.A. (2016). Monocyte activation and cytokine production in Malawian children presenting with P. falciparum malaria. Parasite Immunol..

[19] Bujila I., Schwarzer E., Skorokhod O., Weidner J.M., Troye-Blomberg M., Östlund Farrants A.K. (2016). Malaria-derived hemozoin exerts early modulatory effects on the phenotype and maturation of human dendritic cells. Cell. Microbiol..

[20] Diou J., Tardif M.R., Barat C., Tremblay M.J. (2010). Dendritic cells derived from hemozoin-loaded monocytes display a partial maturation phenotype that promotes HIV-1 trans-infection of CD4+ T cells and virus replication. J. Immunol..

[21] Elliott S.R., Spurck T.P., Dodin J.M., Maier A.G., Voss T.S., Yosaatmadja F., Payne P.D., McFadden G.I., Cowman A.F., Rogerson S.J. (2007). Inhibition of dendritic cell maturation by malaria is dose dependent and does not require Plasmodium falciparum erythrocyte membrane protein 1. Infect. Immun..

[22] Millington O.R., Gibson V.B., Rush C.M., Zinselmeyer B.H., Phillips R.S., Garside P., Brewer J.M. (2007). Malaria impairs T cell clustering and immune priming despite normal signal 1 from dendritic cells. PLoS Pathog..

[23] Scorza T., Magez S., Brys L., De Baetselier P. (1999). Hemozoin is a key factor in the induction of malaria-associated immunosuppression. Parasite Immunol..

[24] Barrera V., Skorokhod O.A., Baci D., Gremo G., Arese P., Schwarzer E. (2011). Host fibrinogen stably bound to hemozoin rapidly activates monocytes via TLR-4 and CD11b/CD18-integrin: a new paradigm of hemozoin action. Blood.

[25] Ortega-Pajares A., Rogerson S.J. (2018). The Rough Guide to Monocytes in Malaria Infection. Front. Immunol..

[26] Schrum J.E., Crabtree J.N., Dobbs K.R., Kiritsy M.C., Reed G.W., Gazzinelli R.T., Netea M.G., Kazura J.W., Dent A.E., Fitzgerald K.A., Golenbock D.T. (2018). Cutting edge: plasmodium falciparum induces trained innate immunity. J. Immunol..

[27] Guha R., Mathioudaki A., Doumbo S., Doumtabe D., Skinner J., Arora G., Siddiqui S., Li S., Kayentao K., Ongoiba A. (2021). Plasmodium falciparum malaria drives epigenetic reprogramming of human monocytes toward a regulatory phenotype. PLoS Pathog..

[28] Netea M.G., Domínguez-Andrés J., Barreiro L.B., Chavakis T., Divangahi M., Fuchs E., Joosten L.A.B., van der Meer J.W.M., Mhlanga M.M., Mulder W.J.M. (2020). Defining trained immunity and its role in health and disease. Nat. Rev. Immunol..

[29] Funes S.C., Rios M., Fernández-Fierro A., Di Genaro M.S., Kalergis A.M. (2022). Trained immunity contribution to autoimmune and inflammatory disorders. Front. Immunol..

[30] Makam P., Matsa R. (2021). "Big three" infectious diseases: tuberculosis, malaria and HIV/AIDS. Curr. Top. Med. Chem..

[31] Tripathi A.K., Sha W., Shulaev V., Stins M.F., Sullivan D.J. (2009). Plasmodium falciparum-infected erythrocytes induce NF-kappaB regulated inflammatory pathways in human cerebral endothelium. Blood.

[32] Griffith J.W., Sun T., McIntosh M.T., Bucala R. (2009). Pure Hemozoin is inflammatory *in vivo* and activates the NALP3 inflammasome via release of uric acid. J. Immunol..

[33] Velagapudi R., Kosoko A.M., Olajide O.A. (2019). Induction of Neuroinflammation and Neurotoxicity by Synthetic Hemozoin. Cell. Mol. Neurobiol..

[34] Loo C.S., Gatchalian J., Liang Y., Leblanc M., Xie M., Ho J., Venkatraghavan B., Hargreaves D.C., Zheng Y. (2020). A genome-wide CRISPR screen reveals a role for the non-canonical nucleosome-remodeling BAF complex in Foxp3 expression and regulatory T cell function. Immunity.

[35] Baxter A.E., Huang H., Giles J.R., Chen Z., Wu J.E., Drury S., Dalton K., Park S.L., Torres L., Simone B.W. (2023). The SWI/SNF chromatin remodeling complexes BAF and PBAF differentially regulate epigenetic transitions in exhausted CD8^+^ T cells. Immunity.

[36] Liao J., Ho J., Burns M., Dykhuizen E.C., Hargreaves D.C. (2024). Collaboration between distinct SWI/SNF chromatin remodeling complexes directs enhancer selection and activation of macrophage inflammatory genes. Immunity.

[37] Ramirez-Carrozzi V.R., Nazarian A.A., Li C.C., Gore S.L., Sridharan R., Imbalzano A.N., Smale S.T. (2006). Selective and antagonistic functions of SWI/SNF and Mi-2beta nucleosome remodeling complexes during an inflammatory response. Genes Dev..

[38] Gamble N., Bradu A., Caldwell J.A., McKeever J., Bolonduro O., Ermis E., Kaiser C., Kim Y., Parks B., Klemm S. (2024). PU.1 and BCL11B sequentially cooperate with RUNX1 to anchor mSWI/SNF to poise the T cell effector landscape. Nat. Immunol..

[39] Lugo-Villarino G., Troegeler A., Balboa L., Lastrucci C., Duval C., Mercier I., Bénard A., Capilla F., Al Saati T., Poincloux R. (2018). The C-type lectin receptor DC-SIGN has an anti-inflammatory role in human M(IL-4) macrophages in response to *Mycobacterium tuberculosis*. Front. Immunol..

[40] Schroder K., Irvine K.M., Taylor M.S., Bokil N.J., Le Cao K.A., Masterman K.A., Labzin L.I., Semple C.A., Kapetanovic R., Fairbairn L. (2012). Conservation and divergence in toll-like receptor 4-regulated gene expression in primary human versus mouse macrophages. Proc. Natl. Acad. Sci. USA.

[41] Baillie J.K., Arner E., Daub C., De Hoon M., Itoh M., Kawaji H., Lassmann T., Carninci P., Forrest A.R.R., Hayashizaki Y. (2017). Analysis of the human monocyte-derived macrophage transcriptome and response to lipopolysaccharide provides new insights into genetic aetiology of inflammatory bowel disease. PLoS Genet..

[42] Andrilenas K.K., Ramlall V., Kurland J., Leung B., Harbaugh A.G., Siggers T. (2018). DNA-binding landscape of IRF3, IRF5 and IRF7 dimers: implications for dimer-specific gene regulation. Nucleic Acids Res..

[43] Shio M.T., Eisenbarth S.C., Savaria M., Vinet A.F., Bellemare M.J., Harder K.W., Sutterwala F.S., Bohle D.S., Descoteaux A., Flavell R.A., Olivier M. (2009). Malarial hemozoin activates the NLRP3 inflammasome through Lyn and Syk kinases. PLoS Pathog..

[44] Pack A.D., Schwartzhoff P.V., Zacharias Z.R., Fernandez-Ruiz D., Heath W.R., Gurung P., Legge K.L., Janse C.J., Butler N.S. (2021). Hemozoin-mediated inflammasome activation limits long-lived anti-malarial immunity. Cell Rep..

[45] Gabay C., Lamacchia C., Palmer G. (2010). IL-1 pathways in inflammation and human diseases. Nat. Rev. Rheumatol..

[46] Vasquez M., Zuniga M., Rodriguez A. (2021). Oxidative stress and pathogenesis in malaria. Front. Cell. Infect. Microbiol..

[47] Adelaja A., Taylor B., Sheu K.M., Liu Y., Luecke S., Hoffmann A. (2021). Six distinct NFκB signaling codons convey discrete information to distinguish stimuli and enable appropriate macrophage responses. Immunity.

[48] Cheng Q.J., Ohta S., Sheu K.M., Spreafico R., Adelaja A., Taylor B., Hoffmann A. (2021). NF-κB dynamics determine the stimulus specificity of epigenomic reprogramming in macrophages. Science.

[49] Raulf M.K., Johannssen T., Matthiesen S., Neumann K., Hachenberg S., Mayer-Lambertz S., Steinbeis F., Hegermann J., Seeberger P.H., Baumgärtner W. (2019). The C-type lectin receptor CLEC12A recognizes plasmodial hemozoin and contributes to cerebral malaria development. Cell Rep..

[50] Al Hamrashdi M., Brady G. (2022). Regulation of IRF3 activation in human antiviral signaling pathways. Biochem. Pharmacol..

[51] Chow K.T., Wilkins C., Narita M., Green R., Knoll M., Loo Y.M., Gale M. (2018). Differential and overlapping immune programs regulated by IRF3 and IRF5 in plasmacytoid dendritic cells. J. Immunol..

[52] Gatchalian J., Liao J., Maxwell M.B., Hargreaves D.C. (2020). Control of stimulus-dependent responses in macrophages by SWI/SNF chromatin remodeling complexes. Trends Immunol..

[53] Xiao G., Jin L.L., Liu C.Q., Wang Y.C., Meng Y.M., Zhou Z.G., Chen J., Yu X.J., Zhang Y.J., Xu J., Zheng L. (2019). EZH2 negatively regulates PD-L1 expression in hepatocellular carcinoma. J. Immunother. Cancer.

[54] Morel K.L., Sheahan A.V., Burkhart D.L., Baca S.C., Boufaied N., Liu Y., Qiu X., Cañadas I., Roehle K., Heckler M. (2021). EZH2 inhibition activates a dsRNA-STING-interferon stress axis that potentiates response to PD-1 checkpoint blockade in prostate cancer. Nat. Cancer.

[55] Lu Y., Liu X., Xie M., Liu M., Ye M., Li M., Chen X.M., Li X., Zhou R. (2017). The NF-κB-responsive long noncoding RNA FIRRE regulates posttranscriptional regulation of inflammatory gene expression through interacting with hnRNPU. J. Immunol..

[56] Liu X., Lu Y., Zhu J., Liu M., Xie M., Ye M., Li M., Wang S., Ming Z., Tong Q. (2019). A long noncoding RNA, antisense IL-7, promotes inflammatory gene transcription through facilitating histone acetylation and switch/sucrose nonfermentable chromatin remodeling. J. Immunol..

[57] Church J., Maitland K. (2014). Invasive bacterial co-infection in African children with Plasmodium falciparum malaria: a systematic review. BMC Med..

[58] Gómez-Pérez G.P., van Bruggen R., Grobusch M.P., Dobaño C. (2014). Plasmodium falciparum malaria and invasive bacterial co-infection in young African children: the dysfunctional spleen hypothesis. Malar. J..

[59] Qiao Y., Giannopoulou E.G., Chan C.H., Park S.H., Gong S., Chen J., Hu X., Elemento O., Ivashkiv L.B. (2013). Synergistic activation of inflammatory cytokine genes by interferon-gamma-induced chromatin remodeling and toll-like receptor signaling. Immunity.

[60] Hu Y., Park-Min K.H., Yarilina A., Ivashkiv L.B. (2008). Regulation of STAT pathways and IRF1 during human dendritic cell maturation by TNF-alpha and PGE2. J. Leukoc. Biol..

[61] Ivashkiv L.B. (2018). IFNγ: signalling, epigenetics and roles in immunity, metabolism, disease and cancer immunotherapy. Nat. Rev. Immunol..

[62] Schoggins J.W. (2019). Interferon-stimulated genes: what do they all do?. Annu. Rev. Virol..

[63] Naigles B., Narla A.V., Soroczynski J., Tsimring L.S., Hao N. (2023). Quantifying dynamic pro-inflammatory gene expression and heterogeneity in single macrophage cells. J. Biol. Chem..

[64] Padmanabhan S., Gaire B., Zou Y., Uddin M.M., Vancurova I. (2022). IFNgamma-induced PD-L1 expression in ovarian cancer cells is regulated by JAK1, STAT1 and IRF1 signaling. Cell. Signal..

[65] Sekrecka A., Kluzek K., Sekrecki M., Boroujeni M.E., Hassani S., Yamauchi S., Sada K., Wesoly J., Bluyssen H.A.R. (2023). Time-dependent recruitment of GAF, ISGF3 and IRF1 complexes shapes IFNalpha and IFNgamma-activated transcriptional responses and explains mechanistic and functional overlap. Cell. Mol. Life Sci..

[66] Ahmed A.U., Williams B.R.G., Hannigan G.E. (2015). Transcriptional activation of inflammatory genes: mechanistic insight into selectivity and diversity. Biomolecules.

[67] Shalek A.K., Satija R., Shuga J., Trombetta J.J., Gennert D., Lu D., Chen P., Gertner R.S., Gaublomme J.T., Yosef N. (2014). Single-cell RNA-seq reveals dynamic paracrine control of cellular variation. Nature.

[68] Mansisidor A.R., Risca V.I. (2022). Chromatin accessibility: methods, mechanisms, and biological insights. Nucleus.

[69] Bullwinkel J., Lüdemann A., Debarry J., Singh P.B. (2011). Epigenotype switching at the CD14 and CD209 genes during differentiation of human monocytes to dendritic cells. Epigenetics.

[70] Lara-Astiaso D., Goñi-Salaverri A., Mendieta-Esteban J., Narayan N., Del Valle C., Gross T., Giotopoulos G., Beinortas T., Navarro-Alonso M., Aguado-Alvaro L.P. (2023). In vivo screening characterizes chromatin factor functions during normal and malignant hematopoiesis. Nat. Genet..

[71] Liao J., Hargreaves D.C. (2024). Coordination of transcription factors and SWI–SNF complexes regulates chromatin priming in developing T cells. Nat. Immunol..

[72] Sugimoto M.A., Vago J.P., Perretti M., Teixeira M.M. (2019). Mediators of the resolution of the inflammatory response. Trends Immunol..

[73] Dersh D., Phelan J.D., Gumina M.E., Wang B., Arbuckle J.H., Holly J., Kishton R.J., Markowitz T.E., Seedhom M.O., Fridlyand N. (2021). Genome-wide screens identify lineage- and tumor-specific genes modulating MHC-I- and MHC-II-restricted immunosurveillance of human lymphomas. Immunity.

[74] Li C., Wang Z., Yao L., Lin X., Jian Y., Li Y., Zhang J., Shao J., Tran P.D., Hagman J.R. (2024). Mi-2β promotes immune evasion in melanoma by activating EZH2 methylation. Nat. Commun..

[75] de Dieuleveult M., Yen K., Hmitou I., Depaux A., Boussouar F., Bou Dargham D., Jounier S., Humbertclaude H., Ribierre F., Baulard C. (2016). Genome-wide nucleosome specificity and function of chromatin remodellers in ES cells. Nature.

[76] Ren G., Ku W.L., Ge G., Hoffman J.A., Kang J.Y., Tang Q., Cui K., He Y., Guan Y., Gao B. (2024). Acute depletion of BRG1 reveals its primary function as an activator of transcription. Nat. Commun..

[77] Bornelöv S., Reynolds N., Xenophontos M., Gharbi S., Johnstone E., Floyd R., Ralser M., Signolet J., Loos R., Dietmann S. (2018). The nucleosome remodeling and deacetylation complex modulates chromatin structure at sites of active transcription to fine-tune gene expression. Mol. Cell.

[78] Pundhir S., Su J., Tapia M., Hansen A.M., Haile J.S., Hansen K., Porse B.T. (2023). The impact of SWI/SNF and NuRD inactivation on gene expression is tightly coupled with levels of RNA polymerase II occupancy at promoters. Genome Res..

[79] Innis S.M., Cabot B. (2020). GBAF, a small BAF sub-complex with big implications: a systematic review. Epigenetics Chromatin.

[80] Otto J.E., Ursu O., Wu A.P., Winter E.B., Cuoco M.S., Ma S., Qian K., Michel B.C., Buenrostro J.D., Berger B. (2023). Structural and functional properties of mSWI/SNF chromatin remodeling complexes revealed through single-cell perturbation screens. Mol. Cell.

[81] Miao D., Margolis C.A., Gao W., Voss M.H., Li W., Martini D.J., Norton C., Bossé D., Wankowicz S.M., Cullen D. (2018). Genomic correlates of response to immune checkpoint therapies in clear cell renal cell carcinoma. Science.

[82] Pan D., Kobayashi A., Jiang P., Ferrari de Andrade L., Tay R.E., Luoma A.M., Tsoucas D., Qiu X., Lim K., Rao P. (2018). A major chromatin regulator determines resistance of tumor cells to T cell-mediated killing. Science.

[83] Ahmed N.S., Gatchalian J., Ho J., Burns M.J., Hah N., Wei Z., Downes M., Evans R.M., Hargreaves D.C. (2022). BRD9 regulates interferon-stimulated genes during macrophage activation via cooperation with BET protein BRD4. Proc. Natl. Acad. Sci. USA.

[84] Matikainen S., Nyman T.A., Cypryk W. (2020). Function and regulation of noncanonical caspase-4/5/11 inflammasome. J. Immunol..

[85] Franco A., Flores-Garcia Y., Venezia J., Daoud A., Scott A.L., Zavala F., Sullivan D.J. (2024). Hemozoin-induced IFN-γ production mediates innate immune protection against sporozoite infection. Microbes Infect..

[86] Östlund Farrants A.K., Blomquist P., Kwon H., Wrange O. (1997). Glucocorticoid receptor-glucocorticoid response element binding stimulates nucleosome disruption by the SWI/SNF complex. Mol. Cell Biol..

[87] Kreft L., Soete A., Hulpiau P., Botzki A., Saeys Y., De Bleser P. (2017). ConTra v3: a tool to identify transcription factor binding sites across species, update 2017. Nucleic Acids Res..

