# Supplementary material for: Malaria-derived hemozoin skews dendritic cell responses to bacterial infections by reducing interferon gene-transcription by SWI/SNF-NuRD
Source: iScience. 2025 Jul 3;28(8):113046. doi: 10.1016/j.isci.2025.113046 (PMC12296537; doi:10.1016/j.isci.2025.113046)
Supplement: Document S1. Figures S1–S6 and Table S1 [file mmc1.pdf]

iScience, Volume *28*

Supplemental information

Malaria-derived hemozoin skews dendritic cell responses to bacterial infections by reducing interferon gene-transcription by SWI/SNF-NuRD

Gintare Lasaviciute, Kanwal Tariq, Anaswara Sugathan, Jaclyn Quin, Mareike Polenkowski, Ioana Bujila, Oleksii Skorokhod, Marita Troye-Blomberg, Eva Sverremark-Ekström, and Ann-Kristin Östlund Farrants

Supplementary Figure S1 - Morphology changes and surface markers expression in moDCs upon HZ exposure, Related to Figure 1.

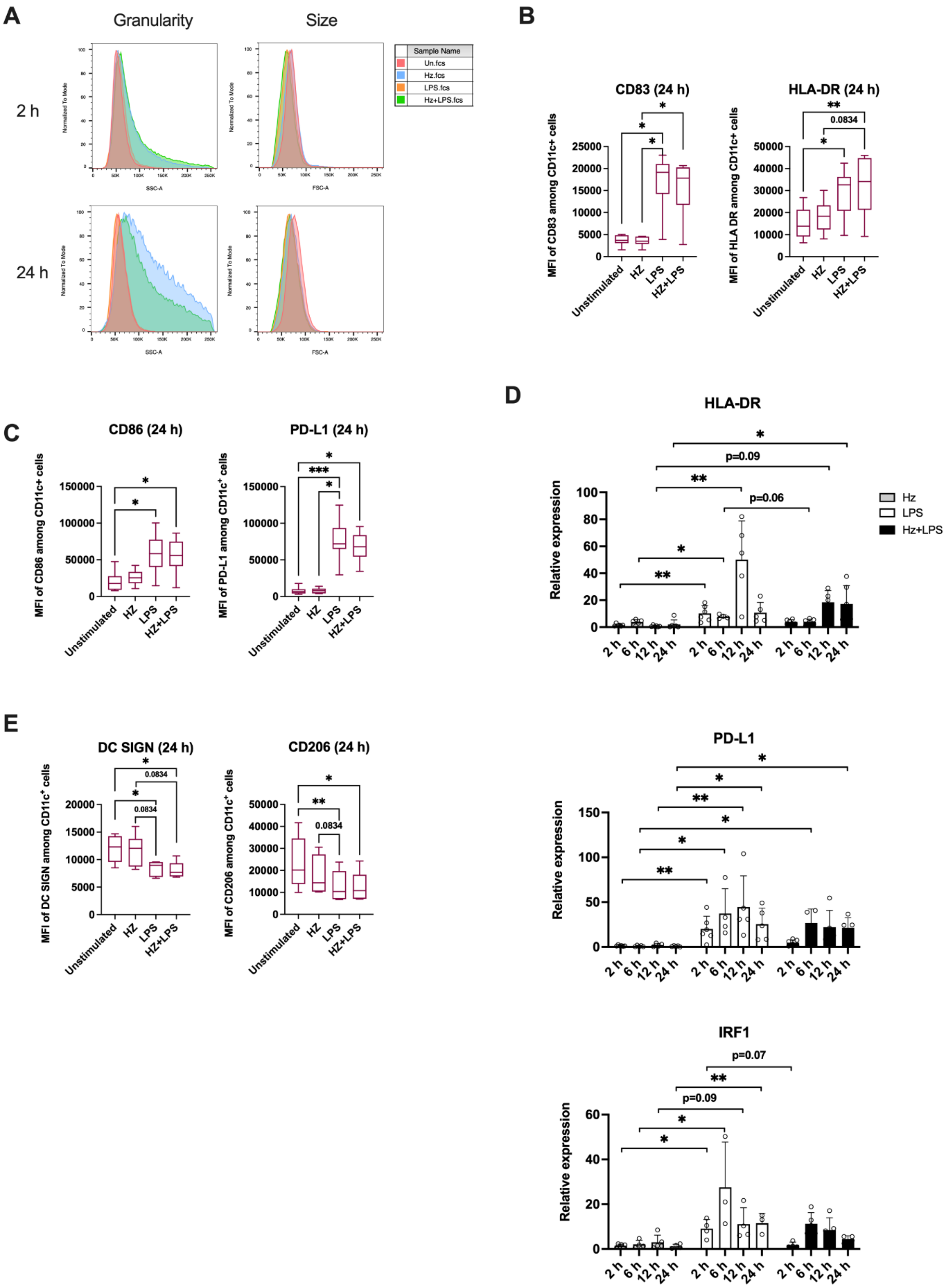

Supplementary Figure S1. Morphology changes and surface markers expression in moDCs upon HZ exposure. (A) Representative flow cytometry histograms showing changes in the size and granularity of moDC after they were exposed to HZ with or without LPS, n=3. **(B)** The mean fluorescent intensity (MFI) of CD83 and HLA-DR, n=6, and **(C)** CD86 and PD-L1, in moDCs after 24 h of exposure, n=6-7. **(D)** Relative mRNA expression of HLA-DR, PD-L1 and IRF1 in moDCs after 2 h, 6 h, 12 h and 24 h of exposures to HZ (grey bars), LPS (white bars), and HZ with LPS (black bars), n=3-6. **(E)** The mean fluorescent intensity (MFI) of DC SIGN and CD206 in moDCs after 24 h of exposure, n=6.

(B, C, E) Boxplots cover data between the 25th and the 75th percentile with median as the central line and whiskers showing min-to-max. Paired Friedman test followed by Dunn´s multiple comparison was used to determine statistical difference, n.s.=p>0.05, *p<0.05, **p<0.01 ***p<0.001.

(D) Bars represent the mean ± standard deviation (SD). A non-parametric Kruskal-Wallis test was used to determine statistical difference n.s.=p>0.05, *p<0.05, **p<0.01.

Supplementary Figure S2 - HZ does not interfere with the production of LPS-induced pro- and anti-inflammatory factors, Related to Figure 2.

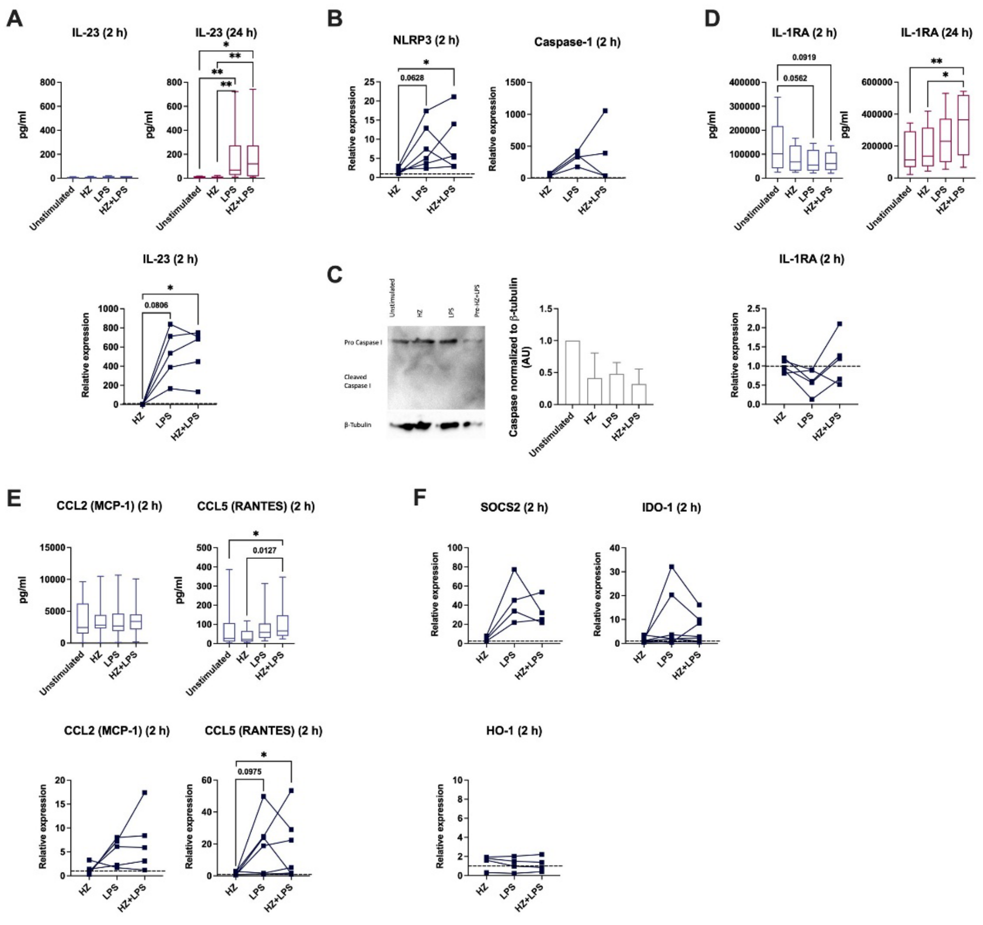

Supplementary Figure S2. HZ does not interfere with the production of LPS-induced pro- and anti-inflammatory factors. (A) The secretion (top graphs) and expression (lower graph), of IL-23 in moDC after 2 h or 24 h exposure, n=4-9. **(B)** Relative mRNA expression of NLRP3 and Caspase-I, n=4-6. **(C)** The representative picture and quantitative analysis of Pro-Caspase-I and cleaved Caspase-I proteins in moDC after short term exposure. Caspase levels were normalized to β-tubulin levels. Bars represent the median with interquartile range n=3. **(D)** The secretion (upper graphs) and expression (lower graph), of IL-1RA in moDCs after 2 h or 24 h exposure, n=5-10. **(E)** The secretion (upper graphs) and expression (lower graphs), of CCL2 and CCL5 after 2 h of exposure, n=5-14. **(F)** Relative mRNA expression of SOCS2, IDO-1 and HO-1, n=4-7. The mRNA levels were measured in all samples after 2 h of exposure.

(A, D, E) Boxplots cover data between the 25th and the 75th percentile with median as the central line and whiskers showing min-to-max.

(A-F) Paired Friedman test followed by Dunn´s multiple comparison was used to determine statistical difference, *p<0.05, **p<0.01.

Supplementary Figure S3 - The surface expression of CLEC12A receptor, IRF3-P, and the recruitment of different transcription factors at the promoters of IL-23, CCL5 and IL-1RA genes, Related to Figure 3.

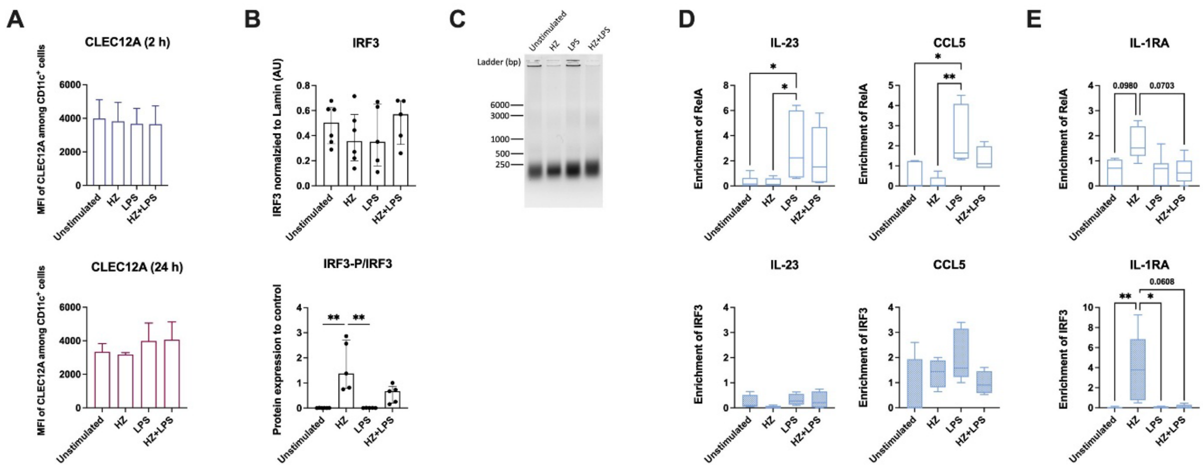

Supplementary Figure S3. The surface expression of CLEC12A receptor, IRF3-P, and the recruitment of different transcription factors at the promoters of IL-23, CCL5 and IL-1RA genes. (A) The mean fluorescent intensity (MFI) of CLEC12A receptor expression in moDCs after 2 h exposure (upper graphs) and 24 h exposure (lower graphs), n=2-4. **(B)** Quantification of immunoblots of IRF3 protein in unstimulated, HZ exposed, LPS-stimulated and LPS and HZ co-exposed moDCs, normalised to the lamin signal on each blot, n=5-6 (top panel), ratio of IRF3-P and IRF3-protein in each sample, n=5-6 (bottom panel). **(C)** Agarose gel, 1%, of DNA-fragments from sonicated chromatin fractions from unstimulated. HZ-exposed, LPS-stimulated, and LPS and HZ co-exposed moDCs for 2 h. The marker is depicted to the right. **(D-E)** The enrichment of RelA (upper graphs) and IRF3 (lower graphs), at the promoters of **(D)** IL-23 and CCL5 genes, n=4-6, and **(E)** IL-1RA gene, n=5-7.

(A-B) Bars represent the median with interquartile range.

(D, E) Boxplots cover data between the 25th and the 75th percentile with median as the central line and whiskers showing min-to-max.

(A-E) Non-parametric Kruskal-Wallis ANOVA test, followed by Dunn´s multiple was comparison was applied to determine significant differences, *p<0.05, **p<0.01.

Supplementary Figure S4 - Chromatin states and the recruitment of histone modifications at the promoters of moDCs genes, Related to Figure 4.

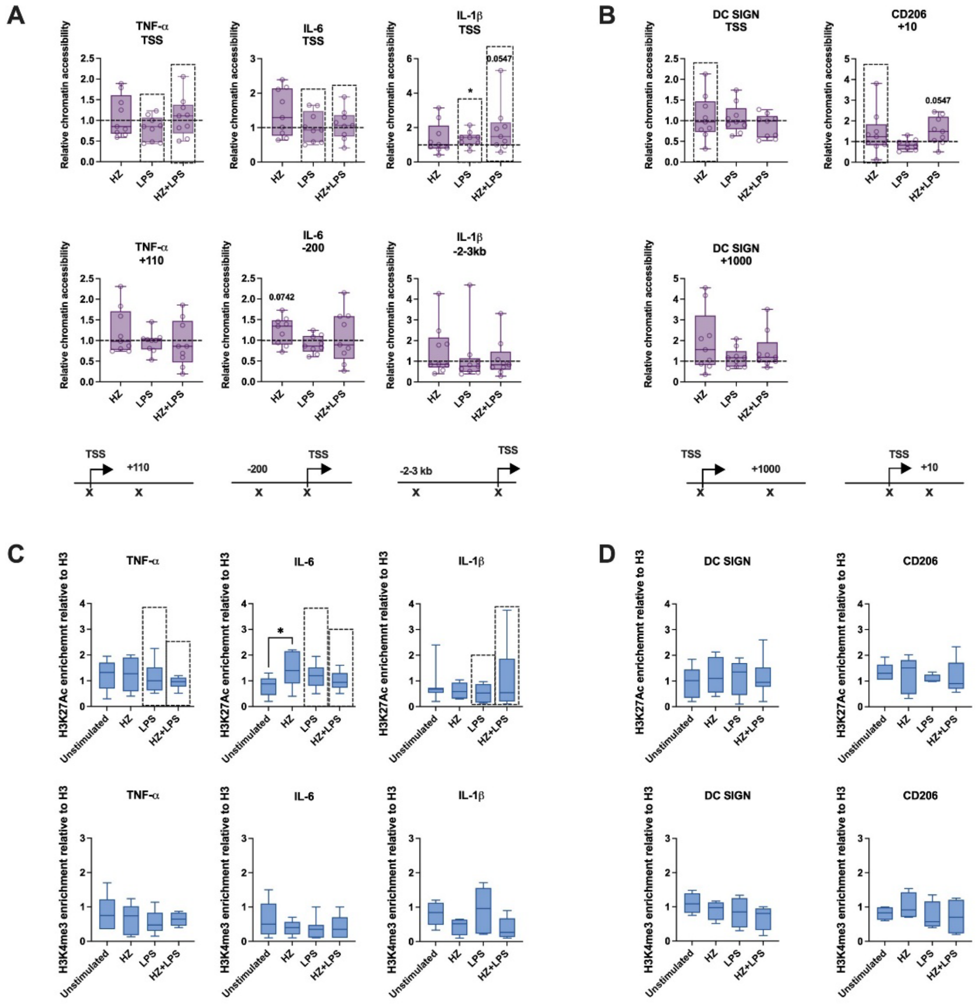

Supplementary Figure S4. Chromatin states and the recruitment of histone modifications at the promoters of moDCs genes. Relative chromatin accessibility at the promoters of **(A)** TNF-α, IL-6 and IL-1β genes, and **(B)** DC SIGN and CD206 genes, n=9. The recruitment of HS27Ac (upper graphs) and H3K4me3 (lower graphs), at the promoters of **(C)** TNF-α, IL-6 and IL-1β genes, n=4-11, and **(D)** DC SIGN and CD206 genes, n=4-10.

(A-D) Boxplots cover data between the 25th and the 75th percentile with median as the central line and whiskers showing min-to-max.

(A-B) Non-parametric Wilcoxon matched-pairs signed rank test was applied to determine significant differences, n.s.=p>0.05.

(C-D) Non-parametric Kruskal-Wallis ANOVA test, followed by Dunn´s multiple was comparison was applied to determine significant differences, *p<0.05.

Supplementary Figure S5 - The recruitment of SWI/SNF complex- and NuRD complex-proteins at the promoters of different moDCs genes, Related to Figure 5.

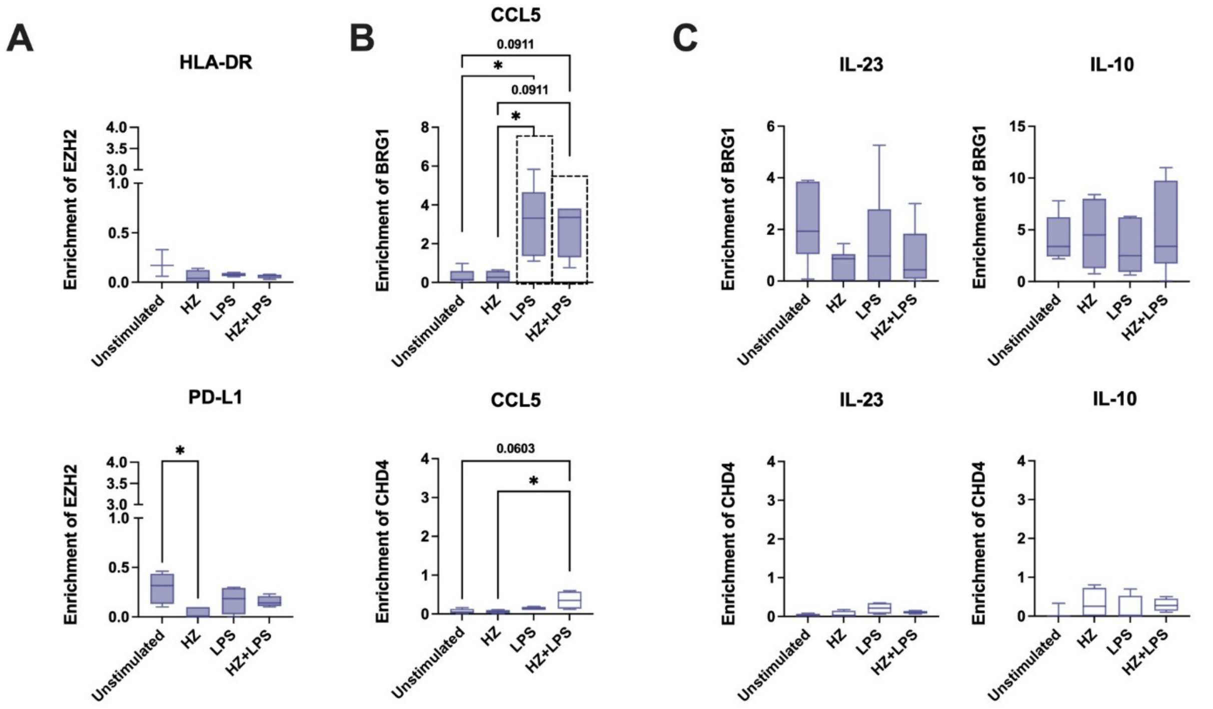

Supplementary Figure S5. The recruitment of SWI/SNF complex- and NuRD complex-proteins at the promoters of different moDCs genes. (A) The enrichment of EZH2 at the promoters of HLA-DR and PD-L1 genes, n=3-5. The enrichment of BRG1 and CHD4 at the promoters of **(B)** CCL5, n=4-5, and **(C)** IL-23 and IL-10, genes, n=3-9.

(A-C) Boxplots cover data between the 25th and the 75th percentile with median as the central line and whiskers showing min-to-max. Non-parametric Kruskal-Wallis ANOVA test, followed by Dunn´s multiple was comparison was applied to determine significant differences, *p<0.05.

Supplementary Figure S6 - Differential recruitment of SWI/SNF complex signature proteins BRD9 and BAF180 /PBMR1 at the CCL5 promoter, Related to Figure 6.

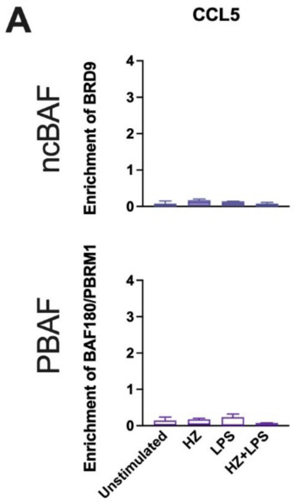

Supplementary Figure 6. Differential recruitment of SWI/SNF complex signature proteins BRD9 and BAF180 /PBRM1 at the CCL5 promoter.

(A) The enrichment of BRD9 (ncBAF), and BAF180/PBRM1 (PBAF) proteins at the promoter of CCL5 gene. Non-parametric Kruskal-Wallis ANOVA test, followed by Dunn´s multiple was comparison was applied to determine significant differences, n.s.=p>0.05, n=5.

Supplementary Table S1. Primer pair sequences.

Gene	Forward (F) / Reverse (R)	Oligonucleotides	References
**cDNA**			
DC SIGN	F	TTG TTG GGC TCT CCT CTG TT	[S1]
	R	AAG TAA CCG CTT CAC CTG GA	
IL-6	F	TAG AGC TTC TCT TTC GTT CCC GGT	[S2]
	R	TGT GTC TTG CGA TGC TAA AGG ACG	
IL-23	F	CTC TGC TCC CTG ATA GCC CT	[S3]
	R	TGC GAA GGA TTT TGA AGC GG	
IL-10	F	GCC TAA CAT GCT TCG AGA TC	[S4]
	R	CTC ATG GCT TTG TAG ATG CC	
CD83	F	ATT CCC TGA AGA TCC GAA AC	[S5]
	R	GAA AAT AAC CAG AGC CAG CA	
CD86	F	GTT GCC TTG AGC AAA AAC AA	[S5]
	R	TGA GAG AGG AAG AGC TGC AA	
PP1A	F	AGA CAA GGT CCC AAA GAC	[S6]
	R	ACC ACC CTG ACA CAT AAA	
CCL2	F	AGTGTCCCAAAGAAGCTGTG	[S5]
	R	GATTCTTGGGTTGTGGAGTG	
CXCL10	F	CCCACGTGTTGAGATCATTG	[S19]
	R	TCCATCACAGCACCGGG	
CXCL8	F	GTTTTTGAAGAGGGCTGAG	Eurofins
	R	TTTGCTTGAAGTTTCACTGG	
TNF-α	F	TGCTTGTTCCTCAGCCTCTT	[S7]
	R	GGTTTGCTACAACATGGGCT	
IL-1β	F	TGTATGTGACTGCCCAAGATG	[S8]
	R	TTAGTGCCGTGAGTTTCCC	
IL-1RA	F	TGTTAACTGCCTCCAGC	Sigma Aldrich
	R	ATACTTGCAAGGACCAAATG	
IL-18	F	GTTTTTGAAGAGGGCTGAG	Eurofins
	R	TTTGCTTGAAGTTTCACTGG	
IL-18BP	F	GGAGGTGCTCAATGAAGGAACC	[S9]
	R	GTGTCCAGCATTGGAAGTGACC	
CCR7	F	ACAGCCTTCCTGTGTGGTTT	[S10]
	R	ATGATGGAGTACATGATAGG	
CCL5	F	AAGTCTCTAGGTTCTGAGC	Sigma Aldrich
	R	TTTTATGGTTGCATTGAGAAC	
HLA DR	F	GATTGGACCTTCCAGACCCTG	Eurofins
	R	ACTTGGGTGCTCCACTTGGCA	
CD206 (MR)	F	AAATTTGAGGGCAGTGAAAG	[S11]
	R	GGATTTGGAGTTTATCTGGTAG	
PD-L1	F	TGCCGACTACAAGCGAATTACTG	[S12]
	R	CTGCTTGTCCAGATGACTTCGG	
NLRP3	F	GATCTTCGCTGCGATCAACA	[S13]
	R	GGGATTCGAAACACGTGCAATA	
HO-1	F	CCCCAACGAAAAGCACATCC	[S14]
	R	AGACAGCTGCCACATTAGGG	
SOCS	F	TGCAAGGATAAGCGGACAGG	[S15]
	R	CAGAGATGGTGCTGACGTGT	
IDO-1	F	TCTGGCCAGCTTCGAGAAAG	[S16]
	R	AGAACTAGACGTGCAAGGCG	

IL-7as	F	TCCTCCCCTGAATCTTCCATTAGTC	[S17]
	R	CCTGGGCAACAGAATGTGACCTT	
FIRRE	F	CTGTGACCTCGCTTCACTTCT	[S18]
	R	GTGGCAAAGAGCAGAAGATAGA	
BRG1 (SMARCA4)	F	GATGTCGATGATGAATATGGC	Sigma Aldrich
	R	ATCTGGTACTGTTTGAGGAC	
Caspase-1	F	CAACTACAGAAGAGTTTGAGG	Sigma Aldrich
	R	AACATTATCTGGTGTGGAAG	
Caspase-4	F	AAGCTCATCCGAATATGGAG	Sigma Aldrich
	R	ATTCTTCATGAGGACAAAGC	
Caspase-8	F	CTACAGGGTCATGCTCTATC	Sigma Aldrich
	R	ATTTGGAGATTTCCTCTTGC	
CXCL9	F	AGGTCAGCCAAAAGAAAAAG	Sigma Aldrich
	R	TGAAGTGGTCTCTTATGTAGTC	
IRF1	F	CTGTGCGAGTGTACCGGATG	Eurofins
	R	ATCCCCACATGACTTCCTCTT	
**ChIP-qPCR**			
TNF promoter	F	CAGGCAGGTTCTCTTCCTCT	[S20]
	R	GCTTTCAGTGCTCATGGTGT	
IL-1β promoter	F	CACTCTTCCACTCCCTCC	[S21]
	R	AGCCTCAAACCCTTCCTC	
IL-6 promoter	F	TAGCCTCAATGACGACCTAAG	[S22]
	R	GTGGGGCTGATTGGAAACCT	
IL-23p19 promoter	F	GGCCTCATTCTGACGTCTTC	[S23]
	R	CTGAAGGACCAGCCAGAGTC	
IL-1RA promoter	F	AGCATATGCAAAAGCCACGG	[S24]
	R	ATGTGCAGAGCCTGTCTTGG	
DC SIGN promoter	F	ATCACAGGGTGGGAAATAA	[S25]
	R	AGTCTTGGTTCCTTGGAGTC	
CD86 promoter	F	GCTCATCTTAACGTCATGTCTG	Qiagen
	R	ATTTAACCCTTTCCTTGCAGTT	
CD83 promoter	F	ACATTGGTGTCGAGTTGGAG	Qiagen
	R	GGTCTT CCTGGGGTGTCTC	
IL-10 promoter	F	CTCCCCAGGAAATCAACT	[S26]
	R	AAAAGCCACAATCAAGGT	
CD206 (MR) promoter	F	CTGCCCAGCTGAAAAGAACT	[S28]
	R	GTTTTTCCAGCCACCCTCAT	
PD-L1 promoter	F	CTTCGAAACTCTTCCCGGTG	[S28]
	R	ACCTCTGCCCAAGGCAGCAA	
IDO-1 promoter	F	ACGGGCAACTTGGTTTCTTC	[S29]
	R	CATGCAAGTCTGTGGTTCACT	
SOCS-1 promoter	F	TCCAGAAGAGAGGGAAACAG	[S30]
	R	GGCGGCTCTCGCGCATGCTC	
HLA-DRA promoter	F	CAAAGGTAGGTGCTGAGGGA	[S31]
	R	TCCATAGGTCTTTTCTCCAATGCT	
CCL5 promoter	F	GTTTTGAGGATACTCCTAACCACGAA	[S17]
	R	ACACACAGCAAATGAATGACAGAGTT	
CXCL8 promoter	F	GCAGAGCTGTGCCTGTTGAT	[S32]
	R	CCTTGGCAAAACTGCACCTG	
CXCL10 promoter	F	AGGAGCAGAGGGAAATTCCGTAAC	[S33]
	R	AACGTGGGGCTAGTGTGCCA	
**ATAC-qPCR**			
ATAC Ad1_noMX	F	AATGATACGGCGACCACCGAGATCTACAC TCGTCGGCAGCGTCAGATGTG	[S34]

DC Sign TSS	R	AGTCTTGGTTCCTTGGAGTC	[S25]
DC Sign +1000	R	TGTGTCCACAGCCAAAAG	[S25]
TNF A TSS	R	CTGGTCCTCTGCTGTCCTTG	[S35]
TNF A+110	R	GCTTTCAGTGCTCATGGTGT	[S20]
IL6 TSS	R	TTCTCTTTCGTTCCCGGTGG	[S36]
IL6-200	R	GTGGGGCTGATTGGAAACCT	[S22]
IL1B TSS	R	TGA AAG CCA TAA AAA CAG CGA GG	-
IL1B-2-3kb	R	AGCCTCAAACCCTTCCTC	[S21]
HLA-DRA -80	R	TTT CTT CTT GGG CGC TCT GT	-
HLA-DRA +160	R	TCCATAGGTCTTTTCTCCAATGCT	[S31]
PDL1 TSS	R	GAG GAA CAA CGC TCC CTA CC	[S37]
PD-L1 -5kb	R	ACCTCTGCCCAAGGCAGCAA	[S28]
CD86 prom	R	ATTTAACCCTTTCCTTGCAGTT	Sigma Aldrich
CD14-150	R	TTAGGCTCCCGAGTCAACAG	Sigma Aldrich
CD14+150	R	AGGTCTAGGAGGCCCCATC	Sigma Aldrich
(CD206) +10	R	GTTTTTCCAGCCACCCTCAT	[S27]

References

[S1] Lech M, Susanti HE, Römmele C, Gröbmayr R, Günthner R, Anders HJ. Quantitative expression of C-Type lectin receptors in humans and mice. Int J Mol Sci. 2012;13(8):10113–31.

[S2] Ghosh CC, Ramaswami S, Juvekar A, Vu H-Y, Galdieri L, Davidson D, et al. Gene-Specific Repression of Proinflammatory Cytokines in Stimulated Human Macrophages by Nuclear IκBα. J Immunol. 2010;185(6):3685–93.

[S3] Shi Q, Yin Z, Zhao B, Sun F, Yu H, Yin X, et al. PGE2 Elevates IL-23 Production in Human Dendritic Cells via a cAMP Dependent Pathway. Mediators Inflamm. 2015;2015.

[S4] Staples KJ, Smallie T, Williams LM, Foey A, Burke B, Foxwell BMJ, et al. IL-10 Induces IL-10 in Primary Human Monocyte-Derived Macrophages via the Transcription Factor Stat3. J Immunol. 2007;178(8):4779–85.

[S5] Bujila I, Schwarzer E, Skorokhod O, Weidner JM, Troye-Blomberg M, Östlund Farrants AK. Malaria-derived hemozoin exerts early modulatory effects on the phenotype and maturation of human dendritic cells. Cell Microbiol. 2016;18(3):413–23.

[S6] Quin JE, Bujila I, Chérif M, Sanou GS, Qu Y, Homann MV, et al. Major transcriptional changes observed in the Fulani, an ethnic group less susceptible to malaria. Elife. 2017;6:1–19.

[S7] Schrum JE, Crabtree JN, Dobbs KR, Kiritsy MC, Reed GW, Gazzinelli RT, et al. Plasmodium falciparum Induces Trained Innate Immunity. J Immunol. 2018;200(4):1243–1248

[S8] Luan L, Patil NK, Gu Y, Hernandez A, Boha JK, Fensterheim BA, Wang J, Xu Y, Enkhbaatar P, Stark R, et al. Comparative Transcriptome Profiles of Human Blood in Response to the Toll-like Receptor 4 Ligands Lipopolysaccharide and Monophosphoryl Lipid A. 2017; :1–16. 8.

[S9] Carbotti G, Barisione G, Orengo AM, Brizzolara A, Airoldi I, Bagnoli M, Pinciroli P, Mezzanzanica D, Centurioni MG, Fabbi M, et al. The IL-18 antagonist IL-18-binding protein is produced in the human ovarian cancer microenvironment. Clin Cancer Res 2013; 19:4611–20.

[S10] Côté SC, Pasvanis S, Bounou S, Dumais N. CCR7-specific migration to CCL19 and CCL21 is induced by PGE2 stimulation in human monocytes: Involvement of EP2/EP4 receptors activation. Mol Immunol 2009; 46:2682–93.

[S11] Schaedlich K, Beier LS, Kolbe J, Wabitsch M, Ernst J. Pro-inflammatory effects of DEHP in SGBS-derived adipocytes and THP-1 macrophages. Sci Rep [Internet] 2021; 11:1–12. Available from: https://doi.org/10.1038/s41598-021-85119-3.

[S12] Wang F, Yang L, Xiao M, Zhang Z, Shen J, Anuchapreeda S, Tima S, Chiampanichayakul S, Xiao Z. PD-L1 regulates cell proliferation and apoptosis in acute myeloid leukemia by activating PI3K-AKT signaling pathway. Sci Rep [Internet] 2022; 12:1–12. Available from: https://doi.org/10.1038/s41598-022-15020-0.

[S13] Guo Q, Wu Y, Hou Y, Liu Y, Liu T, Zhang H, Fan C, Guan H, Li Y, Shan Z, et al. Cytokine

secretion and pyroptosis of thyroid follicular cells mediated by enhanced NLRP3, NLRP1, NLRC4, and AIM2 inflammasomes are associated with autoimmune thyroiditis. Front Immunol 2018; 9.
[S14] Cuitino L, Obreque J, Gajardo-Meneses P, Villarroel A, Crisóstomo N, San Francisco IF, Valenzuela RA, Méndez GP, Llanos C. Heme-Oxygenase-1 Is Decreased in Circulating Monocytes and Is Associated With Impaired Phagocytosis and ROS Production in Lupus Nephritis. Front Immunol 2019; 10:1–16.
[S15] Liu J-P, Ye L, Wang X, Liu J-P, Ho W-Z. Cyclosporin A inhibits hepatitis C virus replication and restores interferon-alpha expression in hepatocytes. Transpl Infect Dis [Internet] 2011; 13:24–32. Available from: https://www.ncbi.nlm.nih.gov/pmc/articles/PMC3624763/pdf/nihms412728.pdf
[S16] Kim N-S, Torrez T, Langridge W. LPS Enhances CTB-INSULIN Induction of IDO1 and IL-10 Synthesis in Human Dendritic Cells. Cell Immunol 2019; 338:32–42.
[S17] Liu X, Lu Y, Zhu J, Liu M, Xie M, Ye M, Li M, Wang S, Ming Z, Tong Q, et al. A Long Noncoding RNA, Antisense IL-7, Promotes Inflammatory Gene Transcription through Facilitating Histone Acetylation and Switch/Sucrose Nonfermentable Chromatin Remodeling. J Immunol 2019; 203:1548–59.
[S18] Lu Y, Liu X, Xie M, Liu M, Ye M, Li M, Chen X-M, Li X, Zhou R. The NF-κB–Responsive Long Noncoding RNA FIRRE Regulates Posttranscriptional Regulation of Inflammatory Gene Expression through Interacting with hnRNPU. J Immunol 2017; 199:3571–82.
[S19] Comet NR, Aguiló JI, Rathoré MG, Catalán E, Garaude J, Uzé G, Naval J, Pardo J, Villalba M, Anel A. IFNα signaling through PKC-θ is essential for antitumor NK cell function. Oncoimmunology 2014; 3:e948705.
[S20] Kleinnijenhuis J, Quintin J, Preijers F, Joosten LAB, Ifrim DC, Saeed S, et al. Bacille Calmette-Guérin induces NOD2-dependent nonspecific protection from reinfection via epigenetic reprogramming of monocytes. Proc Natl Acad Sci U S A. 2012;109(43):1753742.
[S21] Chan C, Li L, McCall CE, Yoza BK. Endotoxin Tolerance Disrupts Chromatin Remodeling and NF-κB Transactivation at the IL-1β Promoter. J Immunol. 2005;175(1):461–8.
[S22] Zimmermann M, Aguilera FB, Castellucci M, Rossato M, Costa S, Lunardi C, et al. Chromatin remodelling and autocrine TNFα are required for optimal interleukin-6 expression in activated human neutrophils. Nat Commun. 2015;6.
[S23] Goodall JC, Wu C, Zhang Y, McNeill L, Ellis L, Saudek V, et al. Endoplasmic reticulum stress-induced transcription factor, CHOP, is crucial for dendritic cell IL-23 expression. Proc Natl Acad Sci U S A. 2010;107(41):17698–703.
[S24] Arts RJW, Blok BA, van Crevel R, Joosten LAB, Aaby P, Benn CS, et al. Vitamin A induces inhibitory histone methylation modifications and down-regulates trained immunity in human monocytes. J Leukoc Biol. 2015;98(1):129–36.
[S25] Bullwinkel J, Lüdemann A, Debarry J, Singh PB. Epigenotype switching at the CD14 and CD209 genes during differentiation of human monocytes to dendritic cells. Epigenetics. 2011;6(1):45–51.
[S26] Fu LH, Ma CL, Cong B, Li SJ, Chen HY, Zhang JG. Hypomethylation of proximal CpG motif of interleukin-10 promoter regulates its expression in human rheumatoid arthritis. Acta Pharmacol Sin [Internet]. 2011;32(11):1373–80. Available from: http://dx.doi.org/10.1038/aps.2011.98
[S27] Tikhanovich I, Zhao J, Bridges B, Kumer S, Roberts B, Weinman SA. Arginine methylation regulates c-Myc– dependent transcription by altering promoter recruitment of the acetyltransferase p300. J Biol Chem. 2017;292(32):13333–44.
[S28] Qin G, Wang X, Ye S, Li Y, Chen M, Wang S, et al. NPM1 upregulates the transcription of PD-L1 and suppresses T cell activity in triple-negative breast cancer. Nat Commun Proj Prim [Internet]. 2020;11(1). Available from: http://dx.doi.org/10.1038/s41467-020-15364-z
[S29] Tian CQ, Chen L, Chen HD, Huan XJ, Hu JP, Shen JK, et al. Inhibition of the BET family reduces its new target gene IDO1 exprefile:///Users/gintarelasaviciute/Documents/Projects/Hz Project/Primers/SOCS-1 ChIP primers.pdfssion and the production of l-kynurenine. Cell Death Dis [Internet]. 2019;10(8). Available from: http://dx.doi.org/10.1038/s41419-019-1793-9
[S30] Travagli J, Letourneur M, Bertoglio J, Pierre J. STAT6 and Ets-1 form a stable complex that modulates Socs-1 expression by interleukin-4 in keratinocytes. J Biol Chem [Internet]. 2004;279(34):35183–92. Available from: http://dx.doi.org/10.1074/jbc.M403223200
[S31] Siegler BH, Uhle F, Lichtenstern C, Arens C, Bartkuhn M, Weigand MA, et al. Impact of human sepsis on CCCTC-binding factor associated monocyte transcriptional response of Major Histocompatibility Complex II components. PLoS One. 2018;13(9):1–17.
[S32] Jentho E, Ruiz-Moreno C, Novakovic B, Kourtzelis I, Megchelenbrink WL, Martins R, Chavakis

T, Soares MP, Kalafati L, Guerra J, et al. Trained innate immunity, long-lasting epigenetic modulation, and skewed myelopoiesis by heme. Proc Natl Acad Sci U S A 2021; 118:1–10.
[S33] Harris DP, Bandyopadhyay S, Maxwell TJ, Willard B, Dicorleto PE. Tumor Necrosis Factor (TNF) Induction of CXCL10 in Endothelial Cells Requires Protein Arginine Methyltransferase 5 (PRMT5) -mediated Nuclear Factor (NF)-kB p65 Methylation*. J Biol Chem 2014; 289:15328–39.
[S34] Buenrostro JD, Giresi PG, Zaba LC, Chang HY, Greenleaf WJ. Transposition of native chromatin for fast and sensitive epigenomic profiling of open chromatin, DNA-binding proteins and nucleosome position. Nat Methods 2013; 10:1213–8.
[S35] Paré JF, Tabasinezhad M, Grossman A, Atallah A, Hindmarch CCT, Tyryshkin K, Siemens DR, Graham CH. Association of Histone H3 Trimethylation in Circulating Monocytes with Lack of Early Recurrence in Patients with Bladder Cancer following BCG Induction Therapy. Bl Cancer 2023; 9:175–86.
[S36] Liu S, Zhao K, Su X, Lu L, Zhao H, Zhang X, Wang Y, Wu C, Chen J, Zhou Y, et al. MITA/STING and its alternative splicing isoform MRP restrict hepatitis B virus replication. PLoS One 2017; 12:1–20.
[S37] Liu X, Swen JJ, Diekstra MHM, Boven E, Castellano D, Gelderblom H, Mathijssen RHJ, Vermeulen SH, Oosterwijk E, Junker K, et al. A genetic polymorphism in ctla-4 is associated with overall survival in sunitinib-treated patients with clear cell metastatic renal cell carcinoma. Clin Cancer Res 2018; 24:2350–2356
